# Integrative multi-omics and drug response profiling of childhood acute lymphoblastic leukemia cell lines

**DOI:** 10.1038/s41467-022-29224-5

**Published:** 2022-03-30

**Authors:** Isabelle Rose Leo, Luay Aswad, Matthias Stahl, Elena Kunold, Frederik Post, Tom Erkers, Nona Struyf, Georgios Mermelekas, Rubin Narayan Joshi, Eva Gracia-Villacampa, Päivi Östling, Olli P. Kallioniemi, Katja Pokrovskaja Tamm, Ioannis Siavelis, Janne Lehtiö, Mattias Vesterlund, Rozbeh Jafari

**Affiliations:** 1 Clinical Proteomics Mass Spectrometry, Department of Oncology-Pathology, Karolinska Institutet, Science for Life Laboratory, Tomtebodavägen 23A, 171 65 Solna, Sweden; 2 Institute of Plant Biology and Biotechnology, University of Muenster, Schlossplatz 7, 48149 Muenster, Germany; 3 Molecular Precision Medicine, Department of Oncology-Pathology, Karolinska Institutet, Science for Life Laboratory, Tomtebodavägen 23A, 171 65 Solna, Sweden; 4 Division of Gene Technology, School of Engineering Sciences in Chemistry, Biotechnology and Health, KTH, Science for Life Laboratory, Tomtebodavägen 23A, 171 65 Solna, Sweden; 5 Department of Oncology-Pathology, Karolinska Institutet, J6:140 BioClinicum, Akademiska stråket 1, 171 64 Solna, Sweden

**Keywords:** Acute lymphocytic leukaemia, Mechanism of action, High-throughput screening, Immune cell death, Proteomics

## Abstract

Acute lymphoblastic leukemia (ALL) is the most common childhood cancer. Although standard-of-care chemotherapeutics are sufficient for most ALL cases, there are subsets of patients with poor response who relapse in disease. The biology underlying differences between subtypes and their response to therapy has only partially been explained by genetic and transcriptomic profiling. Here, we perform comprehensive multi-omic analyses of 49 readily available childhood ALL cell lines, using proteomics, transcriptomics, and pharmacoproteomic characterization. We connect the molecular phenotypes with drug responses to 528 oncology drugs, identifying drug correlations as well as lineage-dependent correlations. We also identify the diacylglycerol-analog bryostatin-1 as a therapeutic candidate in the *MEF2D-HNRNPUL1* fusion high-risk subtype, for which this drug activates pro-apoptotic ERK signaling associated with molecular mediators of pre-B cell negative selection. Our data is the foundation for the interactive online Functional Omics Resource of ALL (FORALL) with navigable proteomics, transcriptomics, and drug sensitivity profiles at https://proteomics.se/forall.

## Abstract (web summary)

Childhood acute lymphoblastic leukemia is characterised by a range of genetic aberrations. Here, the authors use multi-omics profiling of ALL cell lines to connect molecular phenotypes and drug responses to provide an interactive resource of drug sensitivity.

## Introduction

Acute lymphoblastic leukemia (ALL) accounts for ~30% of all cancers in children, making it the most common childhood cancer. Despite the clinical success of broad chemotherapy protocols and allogeneic hematopoietic stem cell transplantation (HSCT)^1,2^, a considerable number of patients (15–20%) continue to experience poor survival outcomes because they are nonresponsive or likely to relapse on standard of care therapeutics^3,4^. Additionally, ~80% of survivors of childhood ALL will experience a post-treatment life-threatening medical event by age 45, an effect hypothesized to result from the severity and duration of ALL treatment protocols^5^. Targeted therapy protocols have demonstrated the potential to reduce the likelihood of post-treatment health complications, improve outcomes, and address resistance to chemotherapy. These targeted approaches have been successfully implemented clinically in protocols giving tyrosine kinase inhibitors to Philadelphia chromosome-positive ALL patients (NCT03911128)^6^. Recently, targeted treatment strategies have expanded to include immunotherapeutic agents such as antibody and chimeric antigen receptor (CAR) -T-cell therapies^7^. However, the adoption of these agents is limited by challenges in production, identifying suitable antigens, and the clinical emergence of adverse events^8,9^. Consequently, there is an immediate need for novel therapeutic modalities for the treatment of high-risk patients and patients who relapse.

Therefore, bridging the gap in therapeutic options for ALL will likely require the development of both pharmacologic and cellular therapies; and this process will depend on the deeper characterization of both the biomarkers and biology of nonresponsive subtypes. While the genetic landscape of childhood ALL has been extensively studied by the implementation of next-generation genomic, transcriptomic, and epigenetic sequencing tools^10,11^, somatic mutations can only partially explain the underlying biology and phenotype, and approximately 25% of childhood ALL patients lack a detectable driving mutation^12^. Although genetic characterizations have improved risk stratification and hope of new molecular targets for therapy, the primary challenge of identifying novel and effective treatments for ALL is achieving more reliable phenotypic stratification that can improve therapeutic response^13^.

Considering that post-transcriptional and post-translational mechanisms have diverse impacts on the protein levels for each cellular component in ways shaped by cellular fitness requirements^14^, the proteome represents an ideal framework for understanding cellular phenotypes. Recent studies have demonstrated a poor correlation between the proteomic and the genetic phenotypes of cancers^15–17^, including childhood ALL^18^. Studies of tumors and human-derived cell lines^19–22^ demonstrated the importance of proteomic characterization, however, our recent characterization of *ETV6-RUNX1* and hyperdiploid ALL^18^ and a study on PDX models^23^ are the only in-depth studies that have used proteomics methods to understand the biology of childhood ALL.

In this work, we provide a proteomics-guided analysis of 49 childhood ALL cell lines, generate a comprehensive resource of biomarkers and drug sensitivities, and identify a potential therapeutic vulnerability to target the *MEF2D-HNRNPUL1* rearranged high-risk subgroup. We also provide a user-friendly web application for exploration of the proteomic, transcriptomic, and drug sensitivity data described in this study, available at https://proteomics.se/forall.

## Results

### In-depth multi-omics profiling of childhood ALL cell lines

**Fig. 1:**
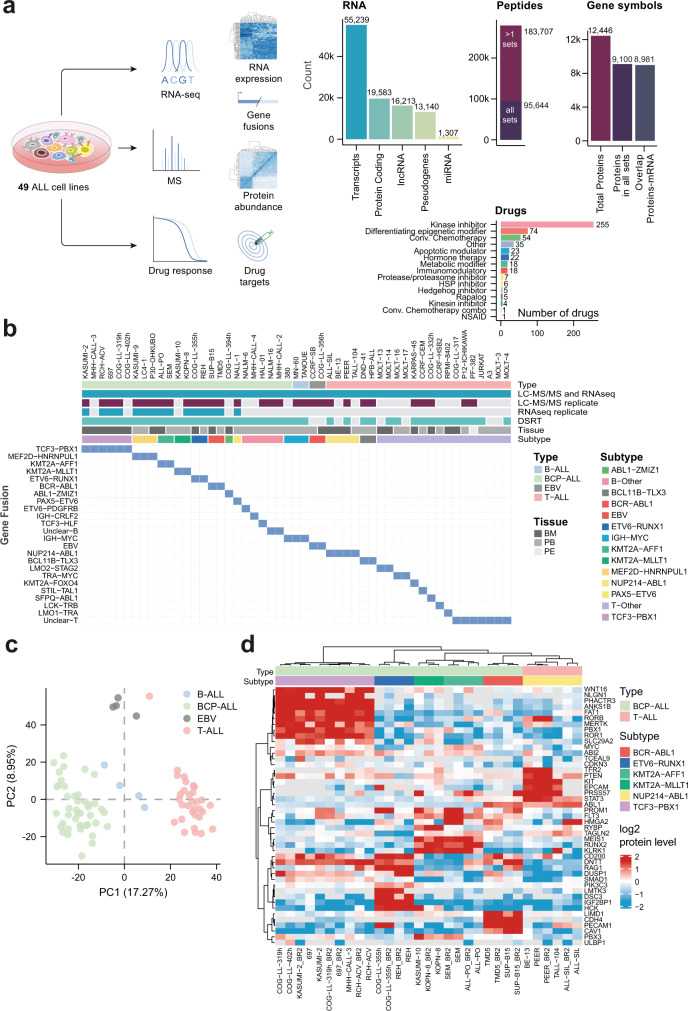
Multi-omics profiling of childhood ALL cell lines. **a** Schematic workflow of childhood ALL cell lines data generation and analyses conducted in this study. Cells were profiled for protein level using mass spectrometry and mRNA expression using RNA-seq. The majority of the cell lines, excluding derivative or sister cell lines (*n* = 43) were scanned for their sensitivity to 528 investigational and oncology drugs. **b** An overview of the most prominent molecular features of the 51 cell lines in this panel. BM bone marrow, PB peripheral blood, PE peripheral effusion, DSRT drug sensitivity and resistance testing. **c** Principal component analysis (PCA) of the highly variable proteins in the 51 cell lines showed distinct clusters corresponding to BCP-ALL (green), immature B-ALL (blue), T-ALL (pink), and EBV-transformed B cells (gray). **d** Cell lines grouped by the most prominent features of the described cytogenetic subtypes using proteomics data. BR biological replicate. Source data are provided as a Source Data File 1.

We performed in-depth profiling by examining protein and RNA levels as well as drug sensitivities (Fig. 1a) of 51 readily available cell lines representing 29 B-lineage and 22 T-lineage cell lines with various cytogenetic backgrounds (Fig. 1b and Supplementary Data 1). Liquid chromatography-mass spectrometry (LC-MS) based protein and peptide quantification for the cell lines and subsequent analyses identified and quantified 279,351 peptides. These were assigned to 13,704 proteins originating from 12,446 genes at a false discovery rate (FDR) of 1%, with a median protein sequence coverage of 46% and an overlap of 9100 proteins (gene symbol-centric) across all the 51 cell lines (Fig. 1a and Supplementary Data 3). Multiplexing was achieved by peptide labeling using tandem mass tags (TMT), and in several of the TMT sets technical and or biological replicates of the SEM cell line were used for the analysis of methodological variability (Supplementary Fig. 1a). Biological replicates for the majority of the cell lines were generated ~1 year apart, where all replicates were grouped together for each cell line (Supplementary Fig. 1b). Transcriptomic profiling was performed using ribosomal depleted total RNA for all 51 cell lines and additionally for 15 replicates with matching proteomic samples (Fig. 1b and Supplementary Data 4) with a minimum and median sequencing depth of 33 M and 47 M paired-end reads, respectively. Fusion analysis was performed using FusionCatcher^24^ for all cell lines and available replicates (Supplementary Data 5). Drug sensitivity and resistance testing (DSRT) was performed for 43 of the cell lines using a panel of 528 approved oncological drugs as well as investigational drugs (Fig. 1a and Supplementary Data 6).

Principal component analysis (PCA) of proteome profiles separated four major lineage-linked groups (Fig. 1c). Cell lines from the T-lineage and B-lineage were clearly separated in this analysis, and the B-lineage cell lines were further distinguished into B-cell Precursor Acute Lymphoblastic Leukemia (BCP-ALL) and B-ALL cell lines. Two cell lines (CCRF-SB and COG-LL-356h) were Epstein-Barr virus (EBV)-transformed B-cell lines, which comprised a fourth phenotypic group. To determine differentially expressed (DE) proteins across cell lines in our panel we performed quantitative proteomics comparison using differential expression quantitative mass spectrometry (DEqMS)^25^ to compare the different cytogenetic groups. At first, we performed quantitative analysis between the B-cell and T-cell lineages excluding the EBV-transformed B-cell lines with subsequent gene set enrichment analysis (GSEA), which correctly indicated B-cell-receptor or T-cell-receptor signaling pathways for each respective lineage (Supplementary Fig. 1c and Supplementary Data 7).

Stratified by cytogenetic background, our dataset was confirmed to reflect enrichment of known markers and pathways. Fusion proteins known to act as leukemia-driving lesions or signatures were enriched in our proteomics dataset, including PBX1, ROR1, and WNT16 for the *TCF3-PBX1* fusion^26^, and ABL1 for *BCR-ABL1*, *NUP214-ABL1*, and *SPFQ-ABL1* fusions (Fig. 1d). Other known markers associated with specific translocations could also be detected, including IGF2BP1 and PIK3C3 for *ETV6-RUNX1*^27,28^ and MEIS1 for *KMT2A*/mixed-lineage leukemia^29^ (Fig. 1d and Supplementary Fig. 1d). In addition, we performed DE analysis of selected subtypes using transcriptomics data from ALL patient samples (EGAD00001002704 and EGAD00001002692, Supplementary Data 8) and compared them with differentially abundant proteins in cell lines with matching cytogenetic subtype which showed excellent agreement between the clinical patient samples and the cell lines (Supplementary Fig. 1e). These results demonstrated that these cell lines retain the molecular signature of clinical ALL patient samples.

### Correlation analysis of ALL proteome and transcriptome

**Fig. 2:**
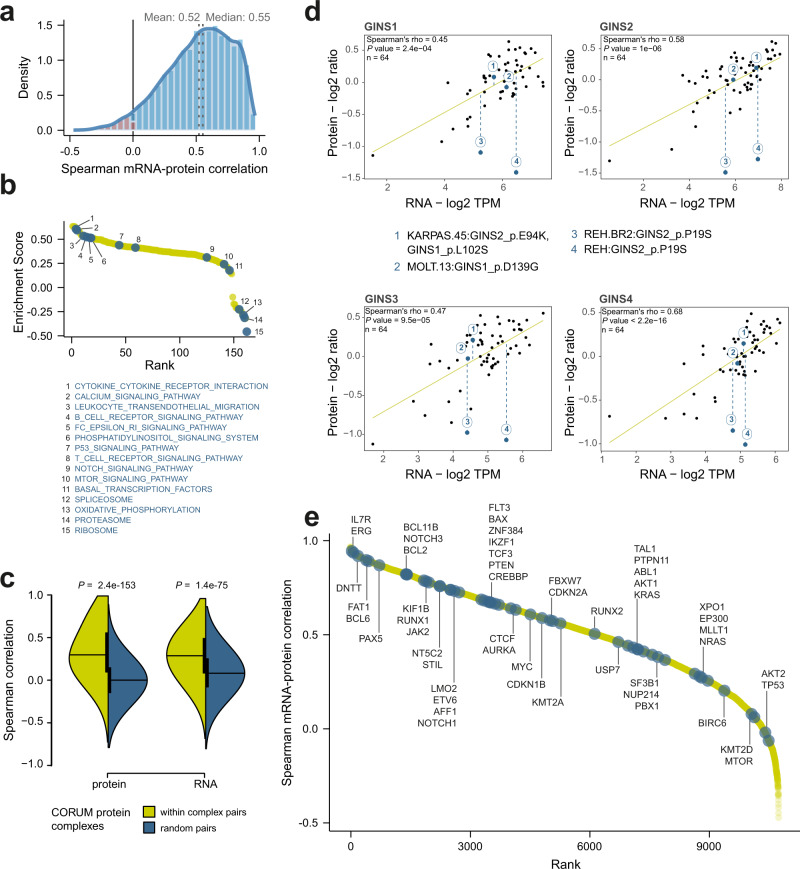
Synergies and discordances of mRNA and protein levels in childhood ALL cell line**s**. **a** Density plot of Spearman rank order of gene symbol-wise correlation of the mRNA-protein levels (*n* = 10,738 RNA-protein pairs), with RNA or protein markers stratified by gene symbol. The mRNA-protein levels were positively correlated for 97% of the pairs across the entire matching panel (*n* = 64 samples), with 78% of the pairs showing significant positive correlation (FDR ≤0.01) assessed by t-distribution adjusted *P* value. **b** GSEA of the mRNA-protein level correlations for KEGG pathways (*n* = 162 ranked pathways) showing that certain specialized signal transduction pathways were enriched among the highly correlating pairs, while pathways associated with the spliceosome, proteasome, and ribosomes belonged to the lowest mRNA-protein correlating pairs. Selected pathways are annotated with blue circles**. c** Comparison of random pairwise correlations (blue) of quantitative proteomics data and mRNA levels across the 49 ALL cell lines and their replicates to known (yellow) interaction pairs (*n* = 31,842) from the CORUM database demonstrating higher correlations at the protein level. The statistical difference was assessed using an unpaired two-sided *t*-test, with *P* values (*p*) indicated. The center line in the violin plots represents the median, the vertical bars represent the first and third quartiles and the curve represents the density curve. **d** Relative log2 ratio protein levels versus log2 TPM (Transcripts Per Million) mRNA levels of Go-Ichi-Ni-San (GINS) complex members across 49 cell lines and their biological replicates. GINS mutations (amino acid substitution) for GINS-complex members are shown for annotated cell lines MOLT-13, KARPAS-45, and REH. Fitted lines were plotted using a linear fit. Dotted lines represent the difference between the actual protein levels and the mRNA-predicted levels. Correlation *P* values are derived from two-sided t-distribution. **e** Ranked mRNA–protein correlations (*n* = 10,738 ranked mRNA-protein pairs) with selected highlighted markers (blue circles) that are frequently involved in or associated with leukemogenesis. Source data are provided as a Source Data File 2.

Cellular phenotypes are controlled by diverse mechanisms that occur at multiple points following transcription, and we sought to quantify the impact of these mechanisms by performing a systems-level analysis of matched proteome and transcriptome profiles (*n* = 64), excluding the two EBV samples. We performed Spearman pairwise correlation for all mRNA-protein pairs (*n* = 8981), and the median correlation coefficient was 0.55 (Fig. 2a). Our previous study on clinical ALL samples^18^ and other studies on solid cancers demonstrated lower Spearman correlation coefficients for this metric^15,16,30^, however, similar results have been reported for cell line studies^19,31^. This could be a result of the clonal derivation of cell lines or owed to the significant technical advantages of working with cell lines compared to the complexity in sample acquisition, preparation, and cellular fitness requirements of clinical samples. To evaluate the functional impact of proteins detected at levels that diverged from their respective mRNA quantification, a list ranked by Spearman correlation coefficient was used to identify enriched KEGG pathways for the highest and lowest correlated pairs. Highly correlated mRNA-protein pairs belonged to specialized signal transduction pathways such as NFKB1 and LCK in B-cell and T-cell receptor signaling pathways, respectively, while poorly correlating pairs belonged to housekeeping functions such as SRSF2 and RPL5 in spliceosomal and ribosomal processes (Fig. 2b).

Low mRNA–protein correlations in macromolecular complexes prompted us to further investigate the functional proteome. We used a dataset of protein complex members obtained from CORUM^32^ to demonstrate that higher pairwise correlations occur between complex components relative to random pairs at the protein level (*P* = 1.0e-153) than at the transcript level (*P* = 1.4e-75) (Fig. 2c), which was supported by examining values for specific complexes (Supplementary Fig. 2a). In line with previous studies^18,33^, we found similar trends for miRNA targeting, subcellular localization, and protein degradation in shaping protein abundance of complex members reflecting the cumulative and complementary effects of post-transcriptional and post-translational mechanisms^34^ (Supplementary Fig. 2b–e and Supplementary Data 9).

In parallel with the above-mentioned general mechanisms, we detected unique molecular fingerprints on protein-mRNA ratios related to mutational status and complex membership (Supplementary Data 9). For the REH cell line, which harbors a *GINS2* mutation (P19S)^35^, protein-mRNA differences for GINS-complex members were significantly larger than differences for corresponding protein-mRNA pairs in other cell lines (average standardized residuals = −5.08, Supplementary Data 9). This suggests that the mutation could have an impact on the regulation or function of the entire protein complex that is only evident at the proteome level. Our data implicate a potential impairment in complex formation, which would lead to collateral degradation of the other GINS-members^36^ (Fig. 2d).

Considering markers that are frequently involved in ALL initiation and progression, *MTOR*, *AKT2*, and *KMT2D* displayed a poor mRNA–protein correlation, and *TP53* showed no correlation (Fig. 2e). This is in line with the previous observations that *KMT2D* truncating mutations impact protein level but not transcript level^37^. Markers from the B-, and T-cell receptor KEGG pathways showed a similar pattern with a wide range of mRNA–protein correlations (Supplementary Fig. 2f, g). These data further support that transcript level analysis allows for imprecision in interpretations of the protein-level abundance of markers.

### Phenotypic and clustering analysis of proteome and transcriptome profiles

**Fig. 3:**
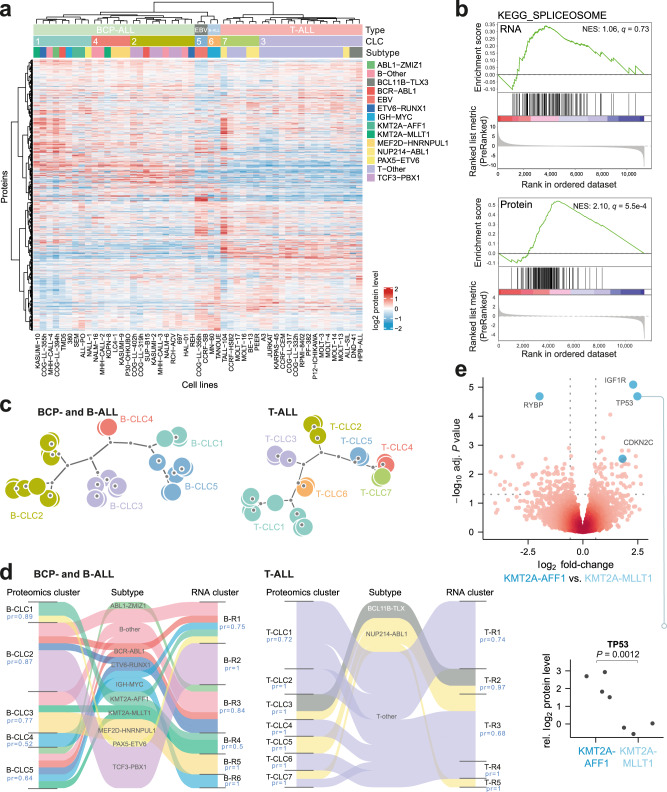
MS-based quantification and clustering of the proteome of childhood ALL cell lines. **a** Heatmap showing the hierarchical clustering of the total overlap of 9100 identified and quantified proteins across 51 cell lines. **b** GSEA of the differentially expressed genes in CLC3 versus the remaining T-ALL cell lines using the proteomics and transcriptomic data showing significant enrichment of the spliceosome in the proteomics data (NES = 2.1, adjusted *P* value *q* = 5.5e-4) but not in the transcriptomic data (NES = 1.06, adjusted *P* value *q* = 0.73). Only one biological replicate from the proteomics data was used to have matching numbers and an unbiased comparison to the transcriptomics data. The significance of NES was assessed by Kolmogorov–Smirnov statistics. **c** Dendrogram of the consensus clustering of the B-lineage and T-lineage separately showing five CLC for B-lineage (B)-CLC) and seven CLC for T-lineage (T-CLC) cell lines. **d** Sankey diagram, showing connection nodes between proteomics CLCs and mRNA-based CLCs, by cytogenetic subtype. The approximately unbiased bootstrapping probability (pr) are indicated for each cluster. **e** Volcano plot of differentially abundant proteins in the *KMT2A-AFF1* cell lines compared to the *KMT2A-MLLT1* cell lines (top panel) using DEqMS and the TP53 levels in *KMT2A-AFF1* cell lines compared to the *KMT2A-MLLT1* cell lines (bottom panel) using two-sided, unpaired *t*-test, *P* value (*p*) is shown in the plot. Source data are provided as a Source Data File 3.

Consensus clustering of the proteome using high variance proteins (*n* = 3282) suggested seven distinct clusters. Hierarchical clustering including all samples and proteins stratified the cell lines into consensus leukemic clusters (CLC) (Fig. 3a, Supplementary Fig. 3a–c, and Supplementary Data 10). Although some clusters included multiple cell lines sharing the same cytogenetic subtype, the majority of the proteomics clusters could not be solely explained by a shared cytogenetic background. CLC1 contained cell lines with *ETV6-RUNX1*, *BCR-ABL1*, *KMT2A-MLLT1*, and *IGH-MYC* gene fusions. CLC2 consisted primarily of *TCF3*-rearranged ALL cell lines (*TCF3-PBX* and *TCF3-HLF*) along with several cell lines with a previously unspecified cytogenetic background. CLC3 contained 16 out of 22 T-ALL cell lines, again with various cytogenetic backgrounds and characterized by G2M checkpoint hallmark, higher spliceosome, and higher E2F target activity when compared to the other six T-ALL cell lines that clustered in CLC7 (Supplementary Data 11).

Given that co-transcriptional regulatory mechanisms have been implicated in the assembly and degradation of the spliceosome^38^, we performed DE analysis on the transcriptomics data between CLC3 and CLC7 followed by GSEA analysis, which failed to recapitulate the upregulation of the spliceosome (Normalized enrichment score (NES) 0.61, adjusted *P* value, *q* = 1.0) (Fig. 3b). Altered splicing profiles and differential splicing have been implicated in drug and therapy resistance and in ALL^39–41^, and these results provide further support for the value of using the proteome to quantify key markers in ALL.

To gain a better resolution on relative differences between the two different lineages, each lineage was clustered separately. This identified five clusters in the B-lineage (B-CLC) and seven clusters in the T-lineage (T-CLC) cell lines (Fig. 3c). Additionally, we performed consensus clustering of the transcriptomics data, which subdivided B- and T-lineages into six and five different subgroups respectively (Fig. 3d). Three of the cell lines with *KMT2A*-rearranged subtypes (*KMT2A-AFF1* and *KMT2A-MLLT1*) were classified into RNA cluster 4 and one into RNA cluster 1, whereas the proteomics clustering divided these into three different proteomic clusters (B-CLC1, B-CLC3, and B-CLC5), demonstrating that the proteomics analysis delivers an additional angle to reveal phenotypic differences, which may be clinically meaningful. We performed DEqMS analysis between the two *KMT2A*-rearranged subtypes and identified p53 among the top upregulated proteins in *KMT2A-AFF1* cell lines (Fig. 3e and Supplementary Data 12) suggesting differences in the p53 regulatory network between the *KMT2A-*rearranged cell lines. Additionally, the two *ETV6-RUNX1* cell lines (REH and COG-LL-355h) were classified into the same RNA cluster (cluster 3) but clustered into different proteomic clusters (B-CLC2 and B-CLC5).

Mechanisms driving B-lineage and T-lineage hematopoiesis are also implicated in leukemogenesis, where lineage markers are used in clinical practice to characterize clinical ALL cases^42^. To evaluate the lineage states of our cell lines, we used established cellular markers of B-cell and T-cell developmental stages^43,44^ to immunotype the BCP-ALL and T-ALL cell lines (Supplementary Fig. 3d). For T-ALL cell lines, we identified that double-negative (DN) and double-positive (DP) differentiation stages were exclusively separated in different clusters. Among the T-ALL clusters, T-CLC1, T-CLC2, and T-CLC3 were composed of cortical DP cell lines or cell lines with unclear immunotyping, and T-CLC5, T-CLC6, and T-CLC7 contained the DN precursor cell lines (Supplementary Fig. 3e, f). Although TAL1 and LYL1 are major subtype markers used in clinical T-ALL stratification, cell lines with these markers were not phenotypically distinguished in our clustering relative to other DN or DP cell lines.

Within the B-cell state assignments, some cytogenetic subtypes appeared to be confined to one B-cell state, while others occupied multiple cell states and proteomic clusters (Supplementary Fig. 3g, h). Uniform B-cell state assignment was identified for *TCF3-PBX1* and *MEF2D-HNRNPUL1* cell lines, with all samples, typed as pro-B or early pre-B respectively, suggesting that these fusions could be linked to an arrested cell state. These fusions were also associated with unified clustering assignments. B-CLC2 and B-CLC3 were defined by cell lines displaying either pro-B or pre-B-lineage traits, and also demonstrated a high relative abundance of the B-lineage maintaining transcription factor PAX5. In contrast, B-CLC1 and B-CLC5 did not appear to be linked by a shared lineage phenotype. However, B-CLC1 demonstrated significant enrichment of FLT3, a hematopoietic trait with pathogenic effects on clinical outcome, commonly associated with leukemia-driving mutations in childhood ALL^45^. The leukemic marker MME (CD10) is also associated with pathogenic patient risk stratification^46^. B-CLC5 contained the cell lines most enriched for this protein (COG-LL-355h, 380, MHH-CALL-4). Additionally, B-CLC5 was characterized by the abundance of the adhesion molecule CEACAM1, which induces a tolerogenic immune environment through interactions with TIM3^47^, suggesting a common retained affinity for oncogenic immunomodulation^48^.

These observations indicate that both conventional lineage and oncogenic traits contribute to proteome-level differences in our cell line panel. Our phenotypic profiling supports current clinical practice in leukemia stratification and suggests that mass spectrometry-based proteomics could be an effective avenue to explore the drivers that contribute to pathogenic phenotypes.

### The drug sensitivity of childhood ALL cell lines to a set of 528 oncology drugs

**Fig. 4:**
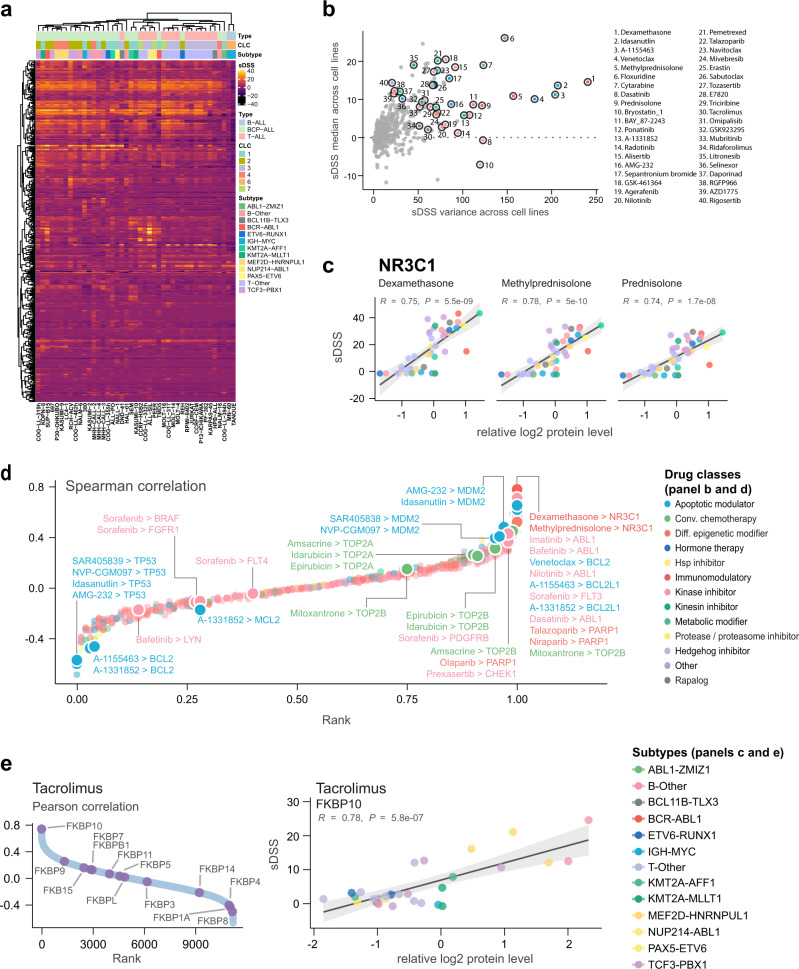
Drug sensitivity and drug target correlations of childhood ALL cell lines. **a** Heatmap depicting the sDSS of the 528 drugs from the DSRT across the 43 tested ALL cell lines. The x-axis is the cell lines ordered by the rank from hierarchical clustering (Pearson ward.D2) and the colors are the individual sDSS for each drug. The legends indicate the CLC and cytogenetic subtype of the cell lines. Higher sDSS indicates a more potent and effective drug relative to normal bone marrow cells. **b** Scatter plot of the median versus variance of the sDSS for each drug across the tested cell lines. Selected drugs are highlighted and colored according to their drug class. **c** The correlation between the sDSS for the glucocorticoids in the DSRT and relative protein level of the glucocorticoid receptor, NR3C1 for the 43 tested ALL cell lines. The x-axis shows the relative log2 protein level of NR3C1 and the y-axis shows the sDSS for each respective cell line (*n* = 43). The Pearson correlation coefficient (*R*) and two-sided t-distribution *P* values (*P*) for the comparison are shown in each plot. The linear regression trendline (black) and its 95% confidence interval (shaded gray area) are shown in the graph. The colors indicate the cytogenetic subtype of the cell lines, as annotated in 4**e**. **d** Scatter plot of the Spearman correlation versus rank for the sDSS of each drug and the relative log2 protein level of its putative protein target. The drugs are colored by drug classes and selected drug–drug target pairs are highlighted. **e** Ranked Pearson correlations of tacrolimus sDSS and highlighted proteins of the FKBP family (purple circles) and a scatter plot of tacrolimus sDSS and FKBP10 levels for each of the 29 tested ALL cell lines where FKBP10 was quantified. The x-axis shows the relative log2 protein level of the protein and the y-axis shows the sDSS for each respective cell line (*n* = 29). The Pearson correlation coefficient (*R*) and two-sided t-distribution *P* values (*P*) for the comparison are shown in each plot. The linear regression trendline (black) and its 95% confidence interval (shaded gray area) are shown in the graph. The colors indicate the cytogenetic subtype of the cell lines. Source data are provided as a Source Data File 4.

There is a demand for new ALL therapeutics to improve outcomes for high-risk patients and address disease relapse. Drug repurposing could help identify promising therapeutics that are safe and clinically viable. The proteome represents a cumulative set of druggable target proteins, therefore we aimed to apply our data to elucidate the processes and proteins that determine sensitivity to drugs. We performed DSRT on 43 of the cell lines (25 BCP-ALL, 16 T-ALL, and 2 B-ALL) against a panel of 528 FDA-approved and investigational oncology drugs at five concentrations. The selective drug sensitivity scores (sDSS) were obtained by normalizing drug sensitivity against drug response of normal bone marrow (BM)^49^ (Fig. 4a and Supplementary Data 13).

Broad-spectrum cytotoxic drugs, e.g., conventional chemotherapy drugs demonstrated high activity in a majority of the tested cell lines (Fig. 4b and Supplementary Fig. 4a). Numerous kinase inhibitors (e.g., targeting Aurora kinases, PLK1, and CHEK1) also demonstrated high activity across most tested cell lines regardless of cytogenetic subtype or lineage (Supplementary Fig. 4a). Notably, many cell lines were sensitive to the p53-MDM2 antagonists (e.g., idasanutlin, SAR405838) except for a group of cell lines, which were consistently resistant to these antagonists (Supplementary Fig. 4b and Supplementary Data 13), among them the *KMT2A-AFF1* cell lines (ALL-PO and SEM) with a suspected deregulated p53 pathway which could be due to mutations in *TP53* or other dysregulation contributed by the gene fusion^50^.

Glucocorticoids (GCs) are components of standard of care combination chemotherapy protocols for childhood ALL^51,52^, and screening of patient cells ex vivo after diagnosis has been shown to predict response or resistance to these therapeutics^53^. Most of the cell lines demonstrated substantial sensitivity to dexamethasone, prednisolone, and methylprednisolone, except for 12 cell lines, among them the REH and JURKAT cell lines (Supplementary Fig. 4a and Supplementary Data 13), which have previously been shown to be resistant to dexamethasone, in agreement with our data^54,55^. Sensitivity to GCs correlated very well with the glucocorticoid receptor (NR3C1) protein abundance (*R* ≥ 0.74, *P* ≤ 1.7e-8) (Fig. 4c), consistent with associations between favorable patient response to GCs and basal expression levels of *NR3C1* in ALL and myelomas^56,57^. Together, these results demonstrate alignment with the activity profiles of clinically and preclinically validated drugs, and that correlated proteins confirmed associated mechanisms.

Using a list of reported putative drug target protein(s) for each drug, we ranked the drug sensitivity-protein abundance Pearson correlations of each drug and its putative drug target to examine the relationship between target abundance and drug response (Fig. 4d). Among the highly ranked drugs in this analysis, several tyrosine kinase inhibitors (e.g., imatinib and bafetinib) and receptor tyrosine kinase inhibitors (e.g., sorafenib and quizartinib) correlated well with protein abundance of their putative targets ABL1 (*R* ≥ 0.71, *P* ≤ 2.64e-12) and FLT3 (*R* ≥ 0.56, *P* ≤ 3.41e-07), respectively (Fig. 4d and Supplementary Fig. 4c, d). Additionally, PARP1 inhibitors talazoparib, niraparib, and olaparib correlated well with PARP1 levels (*R* ≥ 0.43, *P* ≤ 1.64e-04) (Fig. 4d). MDM2 inhibitors positively correlated with MDM2 abundance (*R* ≥ 0.41) while anticorrelating with the target of MDM2 degradation, p53 (*R* ≤ −0.55) (Fig. 4d).

Tacrolimus is a macrolide lactone which was effective (sDSS > 8) for twelve ALL cell lines. This compound is widely used clinically as an immunosuppressant due to its ability to inhibit calcineurin-mediated dephosphorylation of NFAT^58^. Tacrolimus does not target this pathway by direct binding, but rather its activity requires the formation of a complex with FKBP1A, a cis-trans prolyl isomerase belonging to the immunophilin protein family^59^. Despite the crucial role of FKBP1A in this mechanism of action (MoA), we found that the abundance of FKBP1A negatively correlated with sensitivity to tacrolimus (*R* = −0.42, *P* = 0.0046). However, another member of the cis-trans prolyl isomerase family, FKBP10, strongly correlated with tacrolimus sensitivity (*R* = 0.78, *P* = 5.8e-07) (Fig. 4e). FKBP10 is known to bind to tacrolimus, and although previous work has suggested that this interaction affects collagen assembly, the biological impact of the FKBP10/tacrolimus interaction is under-investigated relative to other tacrolimus binding partners^60,61^. This unexpected correlation suggests that FKBP10 merits further study with regard to its binding to tacrolimus and activity profile.

### Mechanisms of drug activity in childhood ALL cell lines

Identification of patients likely to be resistant or responsive to targeted therapy is critical, and understanding the MoA of drugs is an effective first step to achieve this goal. Previous studies have established that omics profiling is an effective method for obtaining mechanistic insight into drug activity. Correlation between mRNA expression and drug sensitivities has been previously used to analyze the MoA of compounds^62^. Reverse-phase protein arrays (RPPA)^20,21^ using ~230 proteins and data-independent acquisition using MS (~3000 proteins)^22^ have also been used for protein drug correlations in cancer cell lines, including seven and two ALL cell lines, respectively. These studies and additional meta-analyses^63^ identified that protein levels had stronger correlations with drug activity than corresponding mRNA levels. To investigate whether these results are confirmed in our childhood ALL cell line panel, we performed pairwise drug sensitivity correlation analysis with our transcriptomics and proteomics data, using drugs with an sDSS of ≥8 in at least two cell lines (*n* = 281) for the analysis. Cumulatively, relative protein abundances correlated to sDSS significantly higher (*P* < 1.45e-20) than protein-coding mRNA levels (Supplementary Fig. 5a), in line with previous observations^20–22^.

**Fig. 5:**
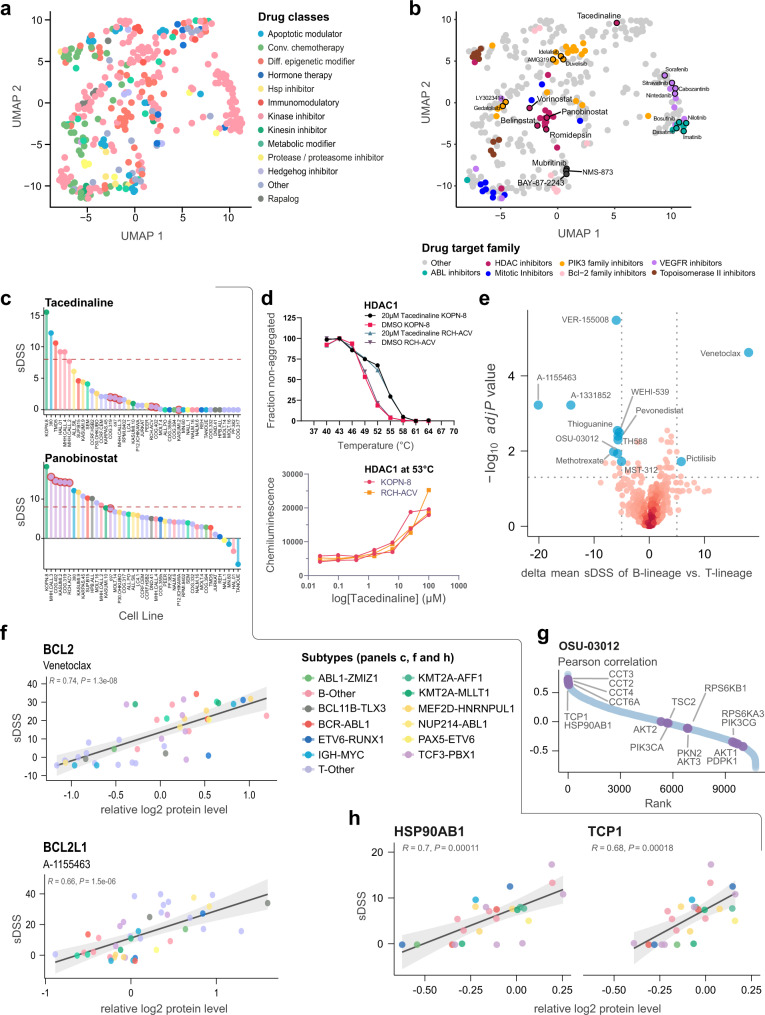
Pharmacoproteomics identifies drug mechanisms of action in childhood ALL cell lines. **a** UMAP of the distribution of class-annotated drugs as a two-dimensional reduction. Drugs were correlated to gene symbol-based protein quantification data based on sDSS, and Pearson correlation scores from this analysis were used to generate the UMAP. Each drug is annotated by its class. **b** UMAP dimensional reduction of each drug, calculated using Pearson correlations between sDSS and protein abundance. Each drug is annotated by a specific target family. **c** sDSS of the tacedinaline (top panel) and panobinostat (bottom panel) across the tested cell lines. The tacedinaline resistant *TCF3-PBX1* fusion cell lines are highlighted with red circles. The red dashed line indicates the selected threshold of sDSS = 8. **d** CETSA derived T_agg_ (aggregation temperature) curves for HDAC1 in KOPN-8 and RCH-ACV cells in the presence of DMSO and 20 μM tacedinaline after 3 h incubation. All data were normalized to the response observed for each treatment condition at the lowest test temperature (top panel). Dose-response CETSA of tacedinaline incubated for 3 h at seven different concentrations ranging from 100 μM to 24 nM at 53 °C based on raw chemiluminescence data from the western blot analysis for HDAC1 in KOPN-8 and RCH-ACV cell lines (bottom panel). Data were provided as two individual data points from two independent experiments (*n* = 2) for each cell line. **e** Volcano plots of differential drug sensitivity between B-lineage and T-lineage ALL cell lines. The cut-off was set at adjusted *t*-test *P* value *q* ≤ 0.02 and the delta mean sDSS between the B-lineage and T-lineage cell lines set at ≥5. Selected drugs are annotated as blue circles. **f** The correlation between the sDSS for venetoclax in the DSRT and relative protein level of BCL2 (top panel) and sDSS for A-1155463 and relative protein level of BCL2L1 (bottom panel) for the 43 tested ALL cell lines. The x-axis shows the relative log2 protein level of the protein and the y-axis shows the sDSS for each respective cell line (*n* = 43). The Pearson correlation coefficient (R) and two-sided t-distribution *P* values (*P*) for the comparison are shown in each plot. The linear regression trendline (black) and its 95% confidence interval (shaded gray area) are shown in the graph. The colors indicate the cytogenetic subtype of the cell lines. **g** Ranked OSU-03012 sDSS and protein Pearson correlations, highlighting members of the TCP1 containing chaperonin complex, HSP90AB1, and PDPK1 interacting proteins from the STRING network database. **h** Scatter plot of OSU-03012 sDSS and relative log2 protein level of HSP90AB1 and TCP1 for each of the BCP-ALL cell lines (*n* = 25). The x-axis shows the relative log2 protein level of the protein and the y-axis shows the sDSS for each respective cell line (*n* = 25). The Pearson correlation coefficient (*R*) and two-sided t-distribution *P* values (*P*) for the comparison are shown in each plot. The linear regression trendline (black) and its 95% confidence interval (shaded gray area) are shown in the graph. Points are labeled by cytogenetic subtype of each represented BCP-ALL cell line. Source data are provided as a Source Data File 5.

For each drug, which was effective (sDSS > 8) for two cell lines at minimum, a matrix of Pearson correlation values for proteins correlated to drug sensitivity was used as input for dimensionality reduction using Uniform Manifold Approximation and Projection (UMAP)^64,65^. (Fig. 5a). We noted close associations in the UMAP space for many drug target families such as ABL1, HDAC, BCL2, and PI3K inhibitors (Fig. 5b). For these drug classes, we examined outliers which were dispersed from their counterparts, and we noted that the selective class I HDAC inhibitor tacedinaline was substantially dispersed from other class I HDAC targeting drugs. Additionally, *TCF3-PBX1* fusion cell lines demonstrated resistance to tacedinaline, despite responding to a majority of other HDAC inhibitors (Fig. 5c and Supplementary Data 13). To study whether the uptake and intracellular concentrations of tacedinaline differ in sensitive and resistant cell lines, we investigated the in-cell target engagement of tacedinaline with HDAC1. Using the cell lines KOPN-8 (*KMT2A-MLLT1*, tacedinaline sensitive) and RCH-ACV (*TCF3-PBX1*, HDACi sensitive, tacedinaline resistant), we monitored HDAC1 engagement using the cellular thermal shift assay (CETSA)^66,67^. This demonstrated that tacedinaline treatment thermostabilized HDAC1 to a similar extent (~4 °C) in both sensitive and resistant cells, and that tacedinaline has equivalent target engagement across a concentration range (Fig. 5d). These observations suggest that despite effective on-target engagement, tacedinaline is distinct from other inhibitors of its class, which confers resistance in cell lines that are otherwise sensitive to HDAC inhibition.

Close associations in UMAP space could also be seen for drugs with different proposed molecular targets, and we confirmed these drug–drug associations by examining correlations between sDSS. Sensitivity to mubritinib, an ERBB2 inhibitor, was associated with HIF1A inhibitor BAY-87-2243 (*R* = 0.87, *P* = 3.2e-14) and VCP/p97 inhibitor NMS-873 (*R* = 0.92, *P* = 5.76e-18) (Fig. 5b). These drugs have a common alternative molecular target, respiratory complex I^68–70^. Additionally, we identified strong positive correlations between sensitivity to these drugs and abundance of proteasomal subunits (Supplementary Fig. 5b, c), indicating that ATP-consuming high protein turnover rates are a shared molecular signature associated with sensitivity. Together, this implicates electron transport chain inhibition leading to reduced ATP availability as a shared MoA for these drugs. Thus, mechanistic insights into unconventional modes of drug activity can be inferred using correlation analysis between drug sensitivity and baseline protein abundance in childhood ALL cell lines.

Studies have previously shown promising efficacy of inhibitors targeting anti-apoptotic BCL2 family members in adult and childhood ALL models^71,72^. In our dataset, the majority of cell lines were very sensitive to broad BCL2 family member inhibitors (i.e., navitoclax, sabutoclax) (Supplementary Fig. 5d, e), but for selective inhibitors of BCL family members, BCP-ALL and T-ALL cell lines were distinguished based on their sensitivity profiles. The BCP-ALL cell lines were very sensitive to venetoclax, a selective BCL2 inhibitor, while the T-ALL cell lines were sensitive to BCL2L1 selective inhibitors A-1155463 and A-1331852 (Fig. 5e). This family of proteins have been shown to determine and restrict lineage choice during hematopoietic differentiation into B- and T-lineages^73^. More recent studies have identified lineage as a contributing factor in predicting the response to these selective agents, but also identified that different dependencies can occur, which could be predicted by abundance of BCL2 or BCL2L1 regardless of lineage^71,72^. In agreement with these studies in our data, the sensitivity of venetoclax and A-1155463 correlated well with abundance of their target proteins, BCL2 (*R* = 0.74, *P* = 1.3e-08) and BCL2L1 (*R* = 0.66, *P* = 1.5e-06), respectively and not exclusively with lineage (Fig. 5f).

The competitive PDPK1 inhibitor and orphan drug OSU-03012 (AR-12) was another targeted drug with lineage specificity (Fig. 5e), where our results indicated excellent sensitivity in most T-ALL cell lines and in a subset of BCP-ALL cell lines. This could reflect lineage-related biology of the PDPK1 kinase, which mediates NOTCH1 signaling during pre-T-cell development^74^. In T-ALL cell lines, the best-correlating protein for OSU-03012 sensitivity was IL9R (*R* = 0.838, *P* = 2.71e-5), a cytokine receptor used as a marker of NOTCH1-dependent developing thymocytes^75^. However, in BCP-ALL cell lines, IL9R was not correlated with sensitivity (*R* = −0.32, *P* = 0.0861), and sensitivity was significantly negatively correlated with PDPK1 protein abundance (*R* = −0.421, *P* = 0.0355). Additionally, no PDPK1 interacting proteins from the STRING network database^76^ correlated with drug activity in BCP-ALL (Fig. 5g), and no significant positive correlation was detected for markers of associated PDPK1 pathways such as mTOR or AKT signaling. Previous work has disputed the designation of OSU-03012 as a PDPK1 inhibitor and identified that cell death following treatment with OSU-03012 occurred via induction of ER-stress signaling^77^. Additional studies identified changes in abundance and stability of heat shock proteins (HSPs) following OSU-03012 treatment and suggested that HSPs were targets of OSU-03012^78^. Our dataset identified a significant correlation with HSP90 (HSP90AB1, Fig. 5h) in BCP-ALL in support of this hypothesis. Additionally, among the most highly correlated proteins were many components of the chaperonin-containing TCP1-complex (CCT-complex) (Fig. 5h), which performs ATP-dependent folding of polypeptide chains. Drug activity via functional interference with this complex would also be consistent with previous data describing induction of ER-stress following drug treatment, and these associations suggest that the CCT-complex could also be studied further as a potential target of OSU-03012. Additionally, assessed by drug–drug correlation, OSU-03012 correlated with the ATP-analog HSP70 family inhibitor VER-155008 in BCP-ALL cell lines (*R* = 0.694, *P* = 0.000119), but not in T-ALL cell lines (*R* = −0.0177, *P* = 0.978). This further suggests that OSU-03012 sensitivity is linked to dependence on chaperonins in BCP-ALL cell lines. Other ongoing clinical, preclinical, and investigational applications of OSU-03012 have also identified the importance of observed changes in chaperone functionality and autophagy induced by drug treatment for several disease indications^79,80^.

To further quantify differences linked to lineage, we used differential correlation analysis (DCA)^81^ applied to the sDSS-protein abundance correlations for the BCP-ALL or T-ALL cell lines (Supplementary Data 14). Here, we identified that the abundance of the thioredoxin TXNDC9 was significantly correlated in opposing directions for numerous clinically relevant chemotherapeutics and topoisomerase inhibitors, including raltitrexed, gemcitabine, and SN-38 (Supplementary Fig. 5f). For BCP-ALLs, increased abundance of this protein correlated with sensitivity, but for T-ALLs, the abundance of this protein was significantly correlated with resistance (Supplementary Fig. 5g). Thioredoxins are known to carry out diverse functions in regulating both the proteasome and redox metabolism, therefore further exploration and characterization of TXNDC9 interactors could reveal insight into lineage-related biological differences. Intriguingly, one well-established binding partner of TXNDC9 is the CCT-complex^82^. Abundance of TXNDC9 was significantly correlated with abundance of all CCT-complex members in BCP-ALL cell lines (e.g., TCP1: *R* = 0.41, *P* = 0.00704), but not in T-ALL cell lines (e.g., TCP1: *R* = 0.07, *P* = 0.72). This suggests that the interaction between TXNCD9 and the CCT-complex is more likely to occur in BCP-ALLs, where TXNDC9 abundance correlates with chemosensitivity rather than chemoresistance.

Together, these differential correlations provide insight into the biology of our cohort which is orthogonal to multi-omics profiling and functionally characterizes important proteins in the context of pharmacologic targeting. Our results demonstrate that integrated proteomics and drug sensitivity analysis is an especially well-suited approach to identify drug-target specificities and to decipher their MoA in ALL. Additionally, our heterogeneous panel of childhood ALL cell lines with varied phenotypes enables a window into the biology of drug response and suggests previously undescribed lineage differences which could have implications in understanding the mechanisms of important therapeutic agents.

### The phenotypic signature and drug sensitivity of MEF2D-rearranged cell lines

Across our analysis of cytogenetic subtype, proteomic clustering, drug sensitivity, and cellular state, we observed striking similarities among a group of cell lines distinguished genetically by a *MEF2D-HNRNPUL1* fusion. *MEF2D* is a member of the myocyte-specific enhancer factor 2 (MEF2) family of transcription factors, which have key roles in a variety of malignancies^83^ and in B-cell development^84^. In the context of childhood ALL, *MEF2D* has been found fused to at least six different fusion partners^85,86^. The *MEF2D*-rearranged cytogenetic subtype is associated with relatively poor clinical outcomes^85–88^, and is also found in adult-onset ALL.

*MEF2D*-rearrangements are associated with increased *HDAC9* levels and MEF2D transcriptional activity. HDAC9 is a class II histone deacetylase that regulates the activity of MEF2 transcription factors^89^. Two studies demonstrated the sensitivity of xenografted and primary culture samples carrying another *MEF2D* fusion, *MEF2D-BLC9* to broad class I HDAC inhibitors^85,88^.

**Fig. 6:**
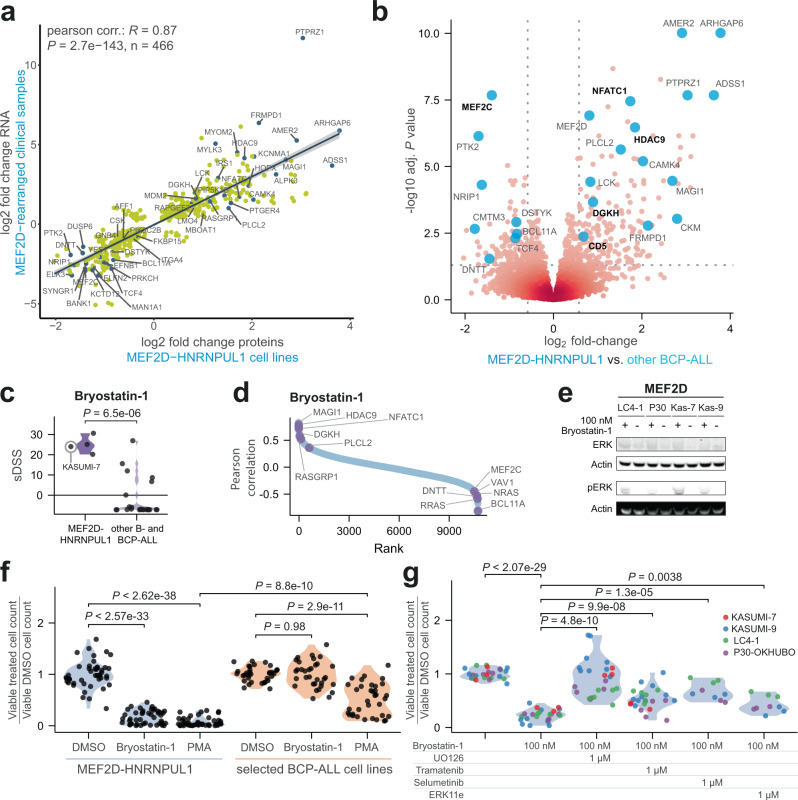
Selective sensitivity of MEF2D-HNRNPUL1 cell lines to bryostatin-1. **a** Fold changes of differentially expressed mRNA using edgeR in 20 clinical patient samples with different *MEF2D*-rearrangements (*MEF2D-BCL9* *n* = 13, *MEF2D-HNRNPUL1* *n* = 4, *MEF2D-CSF1R* *n* = 1, *MEF2D-SS18* *n* = 1, and *MEF2D-DAZAP1* *n* = 1) plotted against differentially abundant proteins in the *MEF2D-HNRNPUL1* cell lines (*n* = 3) using DEqMS. Only differentially expressed mRNA that passed a *P* value ≤0.01 (two-sided *t*-test) are shown in the scatter plot with selected highlighted genes. *N* indicates the number of genes passing this criterion. *R* Pearson correlation coefficient. *P* two-sided t-distribution *P* value. **b** Volcano plot of the differential protein levels in the *MEF2D-HNRNPUL1* cell lines and their replicates (*n* = 6) versus the rest of the BCP-ALL cell lines and replicates (*n* = 37) using DEqMS (spectra counts adjusted *t*-test). The cut-off was set at *P* ≤ 0.05 and the fold change was set to log2(1.5). Selected proteins are highlighted with blue circles. **c** The sDSS of bryostatin-1 in the *MEF2D-HNRNPUL1* cell lines (*n* = 4) compared to the sDSS for the remaining BCP-ALL cell lines (*n* = 24). The two-sided *t*-test *P* value is indicated as (*P*). **d** Ranked bryostatin-1 sDSS and protein level Pearson correlations with selected highlighted proteins in purple. **e** Western blot of ERK1/2 and pERK1/2 (Thr202 and Tyr204) of *MEF2D-HNRNPUL1* fusion cell lines LC4-1, P30-OHKUBO (P30), KASUMI-7 (Kas-7), and KASUMI-9 (Kas-9) after 2 h treatment with 100 nM bryostatin-1. **f** Viable cell quantification normalized to corresponding mean DMSO viable cell count of the four *MEF2D-HNRNPUL1* fusion cell lines, treated with 100 nM of bryostatin-1 or 25 nM PMA. An equal volume of DMSO was used as a control (*n* = 3). BCP-ALL cell lines ALL-PO, REH, RCH-ACV, NALM-6, SUP-B15, COG-LL-355h, COG-LL-394h, and MHH-CALL-2, lacking the *MEF2D-HNRNPUL1* fusion, were subjected to the same treatments. Viable cells were quantified by flow cytometry, excluding zombie aqua dyed non-viable cells. Results are merged from five independent experiments. *P* values were obtained from unpaired two-sided *t*-tests. **g** Viable cell quantification normalized to corresponding mean DMSO viable cell count of the four *MEF2D-HNRNPUL1* fusion cell lines, treated with 100 nM of bryostatin-1 alone or in combination with 1 uM MEK inhibitors UO126, trametinib, or selumetinib. Alternatively, to block ERK directly, 1 uM ERK inhibitor ERK 11e was used, and equal volume of DMSO was used as a control (*n* = 3). Viable cells were quantified by flow cytometry, excluding zombie aqua dyed non-viable cells. Results are merged from three independent experiments. *P* values were obtained from unpaired two-sided *t*-tests. Source data are provided as a Source Data File 6.

*MEF2D-*rearranged leukemias display a distinct gene expression signature^85,86^, which showed substantial overlap and agreement with differentially abundant proteins in the *MEF2D-HNRNPUL1* cell lines (Fig. 6a, Supplementary Fig. 6a, and Supplementary Data 15, 16). Phenotypically, these cell lines clustered together when analyzed by transcriptomics or proteomics, and our cell state assessment immunotyped them uniformly as early pre-B cells (Supplementary Fig. 6c), which is consistent with a recent study of a fourth *MEF2D-HNRNPUL1* fusion cell line (KASUMI-7) derived from an adult patient^90^. DEqMS analysis identified MEF2C and HDAC9 among the top differentially abundant proteins in comparison to the other BCP-ALL cell lines (Fig. 6b, Supplementary Fig. 6b, and Supplementary Data 16), suggesting they may also be sensitive to the broad class I HDAC inhibitors. However, we found no significant difference in sensitivity compared to the other BCP-ALL cell lines (Supplementary Fig. 6d). To investigate this further, we used TMP269, a potent class IIa HDAC inhibitor with an IC50 of 23 nM for HDAC9^91^. Again, we found no inhibitory effect in any of the tested cell lines (Supplementary Fig. 6d). This suggests that HDAC9 may not be a therapeutically targetable vulnerability, at least as leukemic cell-intrinsic toxicity in patients carrying the *MEF2D-HNRNPUL1* fusion.

In contrast to their lack of specific sensitivity to HDAC inhibitors, the three childhood ALL cell lines with the *MEF2D-HNRNPUL1* fusion demonstrated uniquely potent and specific sensitivity to bryostatin-1 relative to other BCP-ALL cell lines (*P* = 6.5e-06). Bryostatin-1 is a diacylglycerol (DAG) analog and protein kinase C (PKC) activator^92^ (Fig. 6c). DSRT analysis of the adult *MEF2D-HNRNPUL1* fusion cell line KASUMI-7 also demonstrated significant sensitivity to bryostatin-1 (sDSS = 24). By assessing proteomic markers with high Pearson correlations to sDSS, we observed a strong correlation between bryostatin-1 toxicity and markers upregulated in the *MEF2D-HNRNPUL1* cell lines (Fig. 6d).

One of the positively correlating markers was RASGRP1 (*R* = 0.54, *P* = 0.0064), calcium- and DAG-regulated guanine nucleotide exchange factor, which has been identified as a direct mediator of sensitivity to negative selection in pre-B cells^93^. Negative selection occurs at specific states of B-cell development, and it influences response to BCR stimulus by triggering programmed cell death after sustained, strong BCR signaling. In addition to limiting self-reactive mature B-cells, this mechanism has also been shown to limit the survival of dysfunctional pre-B cells^94,95^. In cells that are not vulnerable to negative selection, BCR stimulus can also contribute tonic survival signals, and therefore leukemias often demonstrate alterations in B-cell developmental progression that favor survival^96^. As cells that have expressed the pre-B-cell receptor at the early pre-B stage, our *MEF2D* fusion cell lines are at a stage in development that could render them vulnerable to negative selection^94^. In support of an underlying manipulation of this negative selection pathway, we observed that *MEF2D* fusion cells had an enriched abundance of the DAG degrading enzyme DGKH (Fig. 6b).

Negative selection mimicry by pharmacologic methods has been applied to target pre-B-cell leukemia carrying the *BCR-ABL* fusion^97,98^ as well as in B-cell lymphomas^99^, and has also been suggested for treating ALL^100^. The mechanism of negative selection mediated by RASGRP1 acts through calcium-regulated PKC delta activity: calcium-bound PKC delta phosphorylates RASGRP1 at S332, and this phosphorylation allows RASGRP1 to initiate proapoptotic ERK pathway activation^93,99^. Thus, we evaluated whether bryostatin-1, as a DAG analog, works by activating this proapoptotic ERK signaling. Following 2 h of 100 nM bryostatin-1 treatment, we observed a significant increase of the abundance of phosphorylated ERK compared to DMSO-treated controls (Fig. 6e).

To validate that bryostatin-1 acts on-target as a DAG analog, we evaluated whether *MEF2D-HNRNPUL1* cell lines would be sensitive to another DAG analog, Phorbol myristate acetate (PMA). PMA also conferred potent toxicity in *MEF2D-HNRNPUL1* cell lines (Fig. 6f), and this effect was significantly more potent in *MEF2D-HNRNPUL1* cell lines than other tested BCP-ALL cell lines (*n* = 8 BCP-ALL cell lines, *P* = 8.8e-10), suggesting that the on-target effects of bryostatin-1 are replicated by PMA. To further confirm that ERK signaling was a mediator of toxicity, we blocked the ERK pathway (phosphorylation) by using the MEK inhibitors tramatenib, UO126, selumetinib, and the ERK inhibitor ERK 11e. In cells treated with 100 nM bryostatin-1 or 25 nM PMA, concurrent dosing with 1 uM of MEK or ERK inhibitors significantly improved viable cell counts for all tested inhibitors (Fig. 6g and Supplementary Fig. 6e). With the exception of UO126, treatment with 1uM MEK or ERK inhibitors alone resulted in slight but significant reductions in viability for *MEF2D-HNRNPUL1* fusion cell lines, further supporting that drugs targeting ERK phosphorylation specifically benefit viability in the context of correcting DAG analog toxicity (Supplementary Fig. 6f).

These characterizations demonstrate that vulnerability to negative selection at the pre-B stage may be partially conserved in leukemic subsets and targetable by DAG analogs. Thus, further study of this therapeutic strategy in *MEF2D-HNRNPUL1* fusion leukemias is merited. More broadly, this finding illustrates that proteomics coupled with drug screening is an analytical approach that can support robust and replicable identification of molecular mediators of drug toxicity.

## Discussion

We here report an in-depth multi-omics layered analysis of 49 readily available childhood ALL cell lines, quantifying more than 12,000 proteins and 19,000 protein-coding transcripts as well as sensitivity to 528 oncology and investigational drugs. This represents an in-depth proteomic analysis of childhood ALL cell lines covering numerous cytogenetic subtypes, which complements our previous proteogenomic analysis^18^ of the two most common subtypes (i.e., *ETV6-RUNX1* and Hyperdiploid). Our data is amenable to methods of analysis that allow subtype-specific multi-omics phenotyping, and which can be applied to obtain specific mechanisms of drug sensitivity in childhood ALL that are relevant for precision medicine.

We illustrate the potential of this dataset by demonstrating correlation analysis to detect post-transcriptional regulation, which can identify specific processes in individual samples, as in the case of the *GINS2* mutation in the REH cell line which we linked to the degradation of the entire GINS complex. Additionally, we perform in-depth characterization of drug–protein correlations, which we used to identify unexpected indications of secondary targets in the sensitivity mechanism for respiratory complex targeting drugs and for the widely used immunosuppressant tacrolimus. This analysis also identified an unexpected drug activity distinction for the HDAC inhibitor tacedinaline, which was specifically less effective in *TCF3-PBX1* fusion cell lines relative to other inhibitors of its class, and which was independent of engagement to its putative target HDAC1. Further, we examined the biological impact of cellular lineage by examining lineage-specific drug–protein correlations and drug–drug correlations. The PDPK1 inhibitor OSU-03012 had distinct proteins correlated to toxicity for BCP- and T- lineage cell lines indicating alternative mechanisms of action in each lineage. Further, we performed differential correlation analysis, which indicated TXNDC9 in lineage-dependent roles that had opposing correlations to chemosensitivity, and which could be further explored to understand the response to therapy in childhood ALL and lineage-linked biological differences. These analytical approaches are well suited to identify additional findings and to improve the characterization of biological differences and drug response mechanisms.

Genomic profiling has been the major tool utilized for the characterization of childhood ALL. However, protein levels have been demonstrated to have a more direct impact on cellular phenotypes, and our resource and analysis thus provides an improved insight into the biology of leukemia. Our resource complements previous proteomics studies of cancer cell lines^19^ by adding in-depth proteomics profiling of additional 42 childhood ALL cell lines. Overall, our integrative data recapitulated poor mRNA–protein correlations, further highlighting the importance of the addition of proteomic analysis in studying childhood ALL. Although our data is limited to established cell lines which do not cover all known subtypes of childhood ALL, we hope that future studies can cover additional subtypes and close this gap.

Notably, we identified a DAG analog and proposed PKC activator, bryostatin-1, that demonstrated a phenotype- and subtype-specific efficiency in the *MEF2D-HNRNPUL1* cell lines. MEF2D fusions represent ~3.6% of childhood ALL cases, and patients experience 5-year event-free survival of 71%, identifying this patient subset as high-risk of relapse or disease progression in current therapy protocols^85^. Our observations demonstrate that bryostatin-1 activates the proapoptotic PKC/RASGRP1/ERK signaling pathway in early pre-B cells, the progeny stage at which *MEF2D-HNRNPUL1* cell lines are arrested. Bryostatin-1 has successfully completed several phase I and II clinical trials^101^, and this safety and pharmacology profile makes it an excellent candidate for drug repurposing. Due to limitations of in-vitro cell line models, bryostatin-1 and other drugs represented in the study should be further explored in preclinical models and patient samples that better represent relevant microenvironments and cytokine signaling before possible adaptation into the clinic. Additionally, our identification of negative selection vulnerability in *MEF2D-HNRNPUL1* fusion cases represents a biologically therapeutic approach, and as a result of its orthogonal mechanism, it could be especially useful in cases of drug resistance and relapse or pursued in combination therapy. Our results indicate that this drug sensitivity results from on-target DAG analog activity, supported by replicating selective sensitivity using another drug of this class and by rescue experiments. The broad and biologically interconnected landscape of DAG binding proteins should be further characterized to understand the precise mechanism of bryostatin-1 in future studies. Further, we recommend consideration of other orthogonal mechanisms of immune regulation, which were identified to protect against negative selection in pre-B leukemias from other subtypes^97^ and could be relevant in potential resistance mechanisms.

Collectively our proteomics, transcriptomics, pharmacoproteomics analysis, and data portal (https://proteomics.se/forall/) of this childhood ALL cell line panel provide a rich resource for exploration and hypothesis generation.

## Methods

### Cell cultivation

The 49 childhood ALL cell lines and two EBV-transformed B-cell lines used in this study were obtained from Deutsche Sammlung von Mikroorganismen und Zellkulturen GmbH (DSMZ, German Collection of Microorganisms and Cell Cultures, Braunschweig, Germany), from Children’s Oncology Group Childhood Cancer Repository (Lubbock, TX, USA), from American Type Culture Collection (ATCC), Japanese Collection of Research Bioresources Cell Bank (JCRB), European Collection of Authenticated Cell Cultures (ECACC, England) and Banca Biologica e Cell Factory (San Martino, Italy). Roswell Park Memorial Institute (RPMI) 1640 containing 2 mM stable glutamine (l-Ala-l-Gln dipeptide) (AQmedia, Sigma-Aldrich) supplemented with either 10 or 20% fetal bovine serum (FBS, Sigma-Aldrich), 20 mM HEPES (Gibco/Life Technologies), 1 mM sodium pyruvate (Sigma-Aldrich), 1x MEM non-essential amino acids (Sigma-Aldrich), and 1x Penicillin-Streptomycin (Sigma-Aldrich) was preferably used. For a few cell lines that were not growing well in RPMI, we instead used Iscove’s Modified Dulbecco’s Medium (IMDM, Sigma-Aldrich) supplemented with 20% FBS or Nutrient Mixture F-10 Ham supplemented with 10% FBS. Detailed information on the provider of the cell lines and growth media can be found in Supplementary Data 1. Cell lines were grown at 37 °C and 5% CO_2_ to a cell density of ~1–2 million cells/mL. Cells were harvested at 500 × *g* for 3 min and washed twice with Hank’s Balanced Salt Solution (Gibco™ HBSS, no calcium, no magnesium, no phenol red). Aliquots of five million cells were saved for proteomics and transcriptomic analysis. Supernatants were tested negative for mycoplasma by MycoAlert Mycoplasma detection kit (Lonza). All cell lines were authenticated by (short tandem repeat) STR profiling (Eurofins Genomics, Ebersberg, Germany).

### Sample preparation for mass spectrometry

Samples were prepared using a modified version of the spin filter-aided sample preparation (FASP) protocol^102^. A volume equivalent to 200 μg of protein was digested for each sample. Cell pellets were resuspended in a buffer containing 4% SDS, 25 mM HEPES pH 7.6 and 1 mM DTT, and lysed by heating to 95 °C for 5 min and subsequent sonication (Bandelin). Cell debris were removed by centrifugation at 14,000 × *g* for 15 min. The total protein amount was estimated (Bio-Rad DC). For filter-aided sample preparation (FASP), 250 µg of protein sample was mixed with 1 mM DTT, 8 M urea, and 25 mM HEPES pH 7.6 in a centrifugation-filtering unit with a 10-kDa cut-off (Nanosep® Centrifugal Devices with Omega™ Membrane, 10 k). The samples were then centrifuged for 15 min, 14,000 × *g*, followed by another addition of the 8 M urea buffer and centrifugation. Proteins were alkylated by 25 mM IAA, in 8 M urea, 25 mM HEPES pH 7.6 for 10 min, centrifuged, followed by two more additions and centrifugations with 4 M urea, 25 mM HEPES pH 7.6. Protein samples were digested on the filter, first by incubation the samples with Lys-C (Nordic Biolabs (Wako Chemicals GmbH)) overnight at 37 °C and an enzyme:protein ratio of 1:50. In the second digestion step trypsin (Thermo Scientific) was added in 50 mM HEPES at an enzyme:protein ratio of 1:100 and incubated for another 8 h at 37 °C. The addition of trypsin is repeated for a final overnight incubation. After digestion, the filter units were centrifuged for 15 min, 14,000 × *g*, followed by another centrifugation with 50 µL MilliQ water. Peptides were collected and the peptide concentration was determined. The quality of digest was checked for every sample by LC-MS/MS analysis. Peptides were then dried in a speedvac. About 100 µg of peptides were resuspended in 100 mM TEAB pH 8.5 and labeled with isobaric TMT10-tags (Thermo Scientific) according to manufacturer’s instructions, but for 3 h. Labeling efficiency was determined by LC-MS/MS before mixing the samples. In total ten TMT10 sets were prepared, whereas some cell lines were analysed in duplicates to assess the influence of the biological variance. Each TMT set contained a pool for posterior dataset normalization that was composed of lysates of different cell lines and digested together with the individual samples. An overview of the sets and the pool composition is given in table Supplementary Data 1. Individual samples for each TMT set were mixed and were purified by solid-phase extraction using SPE strata-X-C columns (Phenomenex) and dried in a SpeedVac.

### High-resolution isoelectric focusing (HiRIEF) of peptides

The prefractionation method was applied as previously described in ref. ^103^. Sample pools were subjected to peptide IEF-IPG (isoelectric focusing by immobilized pH gradient) in the pI range 3–10 and 3.7–4.9. Dried peptide samples were dissolved in 250 µL rehydration solution containing 8 M urea, and allowed to adsorb to the gel bridge strip by swelling overnight. The 24 cm linear-gradient IPG strips (GE Healthcare) were incubated overnight in an 8 M rehydration solution containing 1% IPG pharmalyte pH 3–10 or 2.5–5, respectively (GE Healthcare). After focusing, the peptides were passively eluted into 72 contiguous fractions with MilliQ water/ 35% acetonitrile (CAN)/ 35% ACN + 0.1% formic acid (FA) using an in-house constructed IPG extractor robotics (GE Healthcare Bio-Sciences AB, prototype instrument) into a 96-well plate (V-bottom, Greiner product #651201), which were then dried in a SpeedVac. The resulting fractions were dried and kept at −20 °C.

### LC-MS/MS runs of the HiRIEF fractions

Online LC-MS was performed using a Dionex UltiMate™ 3000 RSLCnano System coupled to a Q-Exactive-HF mass spectrometer (Thermo Fisher Scientific). Each fraction was subjected to MS analysis. Samples were trapped on a C18 guard-desalting column (Acclaim PepMap 100, 75 μm × 2 cm, nanoViper, C18, 5 µm, 100 Å), and separated on a 50 cm long C18 column (Easy spray PepMap RSLC, C18, 2 μm, 100 Å, 75 μm × 50 cm). The nano capillary solvent A was 95% water, 5% DMSO, 0.1% formic acid; and solvent B was 5% water, 5% DMSO, 95% acetonitrile, 0.1% formic acid. At a constant flow of 0.25 μl min^−1^, the curved gradient went from 2% B up to 40% B in each fraction as shown in Supplementary Data 2, followed by a steep increase to 100% B in 5 min.

FTMS master scans with 60,000 resolution (and mass range 300–1500 m/z) were followed by data-dependent MS/MS (35,000 resolution) on the top five ions using higher-energy collision dissociation (HCD) at 30% normalized collision energy. Precursors were isolated with a 2 m/z window. Automatic gain control (AGC) targets were 1E6 for MS1 and 1E5 for MS2. Maximum injection times were 100 ms for MS1 and 100 ms for MS2. Dynamic exclusion was set to 30 s duration. Precursors with unassigned charge state or charge state 1 were excluded. An underfill ratio of 1% was used.

### Drug sensitivity and resistance testing of ALL cell lines

Drug sensitivity and resistance testing^49^ was performed using ALL cell lines cultured as previously described, with media conditions as noted in Supplementary Data 1. Cells were dispensed (Multidrop Combi, Thermo Fisher Scientific) at a density of 10,000 cells in 25 ul culture media into 384-well tissue culture plates (Corning). The cell lines were tested against 528 drugs and drug combinations in fivefold dilutions across a ten-thousand-fold concentration range. The compounds were diluted in dimethyl sulfoxide or water where appropriate. Acoustic dispenser Echo^®^ was used to plate the drugs (labcyte). Following incubation for 72 h at 37 °C and 5% CO_2_, cell viability (ATP levels) were measured using CellTiter Glo (Promega). Data was collected on an Ensight (Perkin Elmer) system. Data on each plate were normalized to a plate-specific negative control (vehicle) and a positive control (100 umol/L Benzalkonium chloride). Quality control and selective drug-sensitivity score (sDSS) calculation^104^ and data analysis was performed using Breeze (breeze.fimm.fi)^105^. The sDSS are a modified area under the curve-based metric for assessing drug sensitivities. The drug sensitivity to TMP269 was carried out at six different concentrations ranging from 0.0001 to 10 µM at tenfold dilution using DMSO in the same 384-well format as the DSRT described above apart from the data analysis.

### CETSA analysis

#### CETSA temperature range in cells

RCH-ACV and KOPN-8 cells were cultured as previously described, tacedinaline was added to final concentrations of 20 µM to 10 mL suspensions of 1.0 × 10^6^ cells/mL for each cell line, DMSO was added as a vehicle to control samples and incubated for 3 h at 37 °C and 5% CO_2_. Cell suspensions were then centrifuged at 300×*g* for 5 min, the supernatant was discarded and the cells were washed twice with Hank’s Balanced Salt Solution (HBSS, Gibco/Life Technologies). Pelleted cells were resuspended in HBSS and 75 µL cell suspension were aliquoted to 0.2 mL tubes. Samples were then heated in a temperature range of 37–70 °C in a Veriti Thermal Cycler (Applied Biosystems/Thermo Fisher Scientific) for 3 min, followed by 3 min cooling at room temperature and immediate snap-freezing in liquid nitrogen. The cells were then lysed by three repeated freeze-thawing and centrifuged at 21,000×*g* for 30 min at 4°C. The cleared supernatants were transferred to new tubes, denatured in LDS sample buffer (Thermo Fisher Scientific), and analyzed by western blotting.

#### CETSA dose-response in cells

RCH-ACV and KOPN-8 cells were cultured as previously described, tacedinaline was added to final concentrations of 100, 25, 6.25, 1.56, 0.39, 0.098, 0.024 µM and DMSO in 100 µL aliquots of 1.0 × 10^6^ cells/mL respectively and incubated at 37 °C and 5% CO_2_ for 3 h. Two replica experiments for each cell line were performed. Cells were then heated at a constant temperature of 53 °C for 3 min in a Veriti Thermal Cycler (Applied Biosystems/Thermo Fisher Scientific) followed by 3 min cooling at RT and immediate snap-freezing in liquid nitrogen. The cells were then lysed by three repeated freeze-thawing and centrifuged at 21,000×*g* for 30 min at 4 °C. The cleared supernatants were transferred to new tubes, denatured in LDS sample buffer (Thermo Fisher Scientific), and analyzed by western blotting.

### Western blotting

Cells were lysed in Cell Signaling Technologies lysis buffer supplemented with protease and phosphatase inhibitors (Halt™ Protease and Phosphatase Inhibitor Single-Use Cocktail, Thermo Fisher Scientific). Protein concentrations were determined using Bio-Rad DC assay (Bio-Rad). Proteins were denatured in LDS sample buffer (Thermo Fisher Scientific), resolved by SDS-PAGE using NuPAGE™ 4 to 12%, Bis-Tris Gel (Invitrogen™, Thermo Fisher Scientific) and NuPAGE MES SDS Running Buffer (Invitrogen™, Thermo Fisher Scientific), and transferred to Nitrocellulose membranes (Invitrogen™, Thermo Fisher Scientific). SeeBlue™ Plus2 Pre-stained Protein Standard was used as protein ladder (Invitrogen™, Thermo Fisher Scientific). Afterward, the membranes were blocked with 5% nonfat dry milk in TBST (Thermo Fisher Scientific) and incubated with primary antibodies for the appropriate target. Following overnight primary incubation at 4 °C, blots were rinsed using TBST and incubated with the appropriate horseradish peroxidase (HRP)-conjugated secondary antibodies (lot 3208198, Millipore, cat no. AP127P for mouse primary ab and SCBT (sc-2005) for rabbit primary ab used at a dilution of 1:5000). All antibody incubations were diluted in 5% nonfat dry milk in TBST. Protein bands were developed with Clarity ECL Substrate Chemiluminescent HRP substrate (Bio-Rad) in an iBright CL1000 Imaging System (Invitrogen™, Thermo Fisher Scientific). Bands were quantified using the ImageJ software version 1.5Oi and iBright Analysis Software version 4.0.1 (Thermo Fisher Scientific). HDAC1 (Thermo Fisher Scientific, cat. No PA1-860, RRID:AB_2118091, 1:1000 dilution), Phospho-ERK1/2 (Thermo Fisher Scientific, cat. No 14-9109-80, RRID:AB_2572925, 1:1000 dilution), ERK1/2 (Thermo Fisher Scientific, cat. No 13-6200, RRID:AB_2533024, 1:1000 dilution), b-actin (Santa Cruz Biotechnology Cat# sc-47778 HRP, RRID:AB_2714189, 1:500) antibodies were used for Western blot to detect corresponding targets.

### Cell viability assessment by flow cytometry

Cell lines were cultured and diluted to plating density in RPMI 1640 (AQmedia, Sigma-Aldrich) supplemented with 10% fetal bovine serum (FBS, Sigma-Aldrich), 20 mM HEPES (Gibco/Life Technologies), 1 mM sodium pyruvate (Sigma-Aldrich), 1x MEM non-essential amino acids (Sigma-Aldrich), and 1x Penicillin-Streptomycin (Sigma-Aldrich). All cell lines were diluted to a plating density of 500k cells/mL. Cells were treated with soluble compounds at the stated concentrations for 72 h in standard tissue culture incubation conditions (37 °C, 5% CO_2_) in a 96-well sterile tissue culture plate (Corning). All drug treatments and DMSO controls were brought to the same relative DMSO volume of 1:200. Following treatment, non-viable cells were stained using 1:500 Zombie Aqua Live Dead stain (Thermo Fisher), diluted in PBS (Invitrogen), and added directly to the plated cells (1:2 volume). Cell staining was performed for 1.5 h on ice, and during staining and all subsequent steps, cells were protected from light using aluminum foil. Viable cell counts were obtained using a BD Biosciences LSRFortessa flow cytometer, and cells were collected in equal volumes per well using the high throughput sampler (HTS) plate reader. Gating (Supplementary Fig. 7) and quantification was performed using the BD FacsDiva software, and gates were optimized to exclude noise by forward scatter area/side scatter area (FSC-A/SSC-A), to exclude doublets by forward scatter area/forward scatter height (FSC-A/FSC-H), and to exclude dead cells positive in the BV510 channel. Drugs used in these experiments were: bryostatin-1 (Chem Cruz), phorbol 12-myristate 13-acetate (PMA) (Sigma-Aldrich), trametinib (Cayman chemical), selumetinib (Selleckchem), ERK 11e (Tocris Bioscience), and UO126 (Cayman chemical).

### Analysis of LC-MS/MS runs

Orbitrap raw MS/MS files were converted to mzML format using msConvert from the ProteoWizard tool suite^106^. Spectra were then searched using MSGF + (v10072)^107^ and Percolator (v2.08)^108^, where search results from eight subsequent fractions were grouped for Percolator target/decoy analysis. All searches were done against the human protein subset of Ensembl 99 in the Galaxy platform^109^. MSGF + settings included precursor mass tolerance of 10 ppm, fully-tryptic peptides, maximum peptide length of 50 amino acids, and a maximum charge of 6. Fixed modifications were TMT-10plex on lysines and peptide N-termini, and carbamidomethylation on cysteine residues, a variable modification was used for oxidation on methionine residues. Quantification of TMT-10plex reporter ions was done using OpenMS project’s IsobaricAnalyzer (v2.0)^110^. PSMs found at 1% FDR (false discovery rate) were used to infer gene identities.

Protein quantification by TMT-10plex reporter ions was calculated using TMT PSM ratios to the entire sample set (all ten TMT channels) and normalized to the sample median. The median PSM TMT reporter ratio from peptides unique to a gene symbol was used for quantification. Protein FDR were calculated using the picked-FDR method using gene symbols as protein groups and limited to 1% FDR. The eight technical replicates of the SEM cell lines were combined by taking the protein-wise median levels.

### RNA sequencing and transcriptome analysis

Total RNAs were extracted from aliquots harvested at the same time point as proteomics samples using the RNeasy Mini Kit (Qiagen), following manufacturer’s instructions using the option of adding β-mercaptoethanol in the lysis step and using DNAase. RNA concentration and quality were determined using Qubit and RNA Assay kit (Thermo Fisher Scientific) and Bioanalyzer with RNA Nano Chips (Agilent Technologies). RNA libraries were prepared with TruSeq Stranded total RNA RiboZero Kit (Illumina) for ribosomal depletion at the sequencing facilities. Sequencing of the libraries (Paired-end 2 × 150 bp) were performed in three different batches by the NovaSeq6000 S2 platform (Illumina) at the National Genomics Infrastructure (NGI) in Stockholm and SNP&SEQ Technology Platform in Uppsala. Basic quality control of the sequencing data and reads was performed by the facilities using a standard quality control pipeline (average quality per base = 36 ± 0.7) (https://github.com/NationalGenomicsInfrastructure/ngi_pipeline). The reads were preprocessed for an adapter and quality-based trimming using cutadapt^111^ and then mapped to the human reference genome GRCh38 (gencode.v31.p12 primary assembly) using STAR aligner^112^ with enabled chimeric reads detection. All samples showed a good percentage of uniquely mapped reads (average 91%). The mapped reads were summarized/quantified at the gene level using featureCounts^113^ using gencode.v31 comprehensive gene annotation. The combined counts of all samples were filtered and adjusted for technical variation due to the sequencing batches using the ComBat-seq method implemented in the sva R package^114^. The adjusted genes counts were normalized using the edgeR package^115^ using the Trimmed Mean of M-values (TMM) method^116^. Transcriptomic data for a total of 417 leukemia samples including 18 cases with MEF2D-rearrangements from pediatric patients were obtained from The European Genome-phenome Archive (EGA) (Dataset ID: EGAD00001002704 and EGAD00001002692) after Data Access Agreement (DAC) approval from St. Jude Children’s Research Hospital - Washington University Pediatric Cancer Genome Project Steering Committee. Two additional RNA-seq samples of MEF2D-HNRNPUL1 subtype were obtained from the Shanghai Institute of Hematology (SIH) and were added to the St. Jude cohort and processed accordingly using the same RNA-seq analysis pipeline. The reads from bam files were re-aligned to gencode.v31.p12 human genome then the processing steps and annotation files were used identical to the cell lines RNA-seq data processing steps. STAR mapping statistics summary of 419 clinical patient samples are presented in Supplementary Data 8.

### Fusion analysis of transcriptomics data

FusionCatcher v1.33^24^ was implemented to detect fusions from raw fastq files of the 51 cell lines samples and biological replicate runs (*n* = 66) using Ensembl database v102 of human genome GRCh38. FusionCatcher was run using default parameters where three aligners (STAR, BLAT, and Bowtie2) are utilized in combination for detection of potential fusion gene pairs. Genes which showed too many fusion partners (above 99% quantile of a number of partners) were filtered out. A matrix of the (counts per million) CPM of all of the uniquely spanned reads for detected fusions in the cell lines using FusionCatcher are presented in Supplementary Data 5.

### Differential expression (DE) analysis of transcriptomics data

RNA-based differential expression analyses were performed using edgeR (v.3.32.1)^115^. FeatureCounts was used to assemble a raw counts matrix, and edgeR used this counts matrix to perform differential expression analysis based on the negative binomial distribution.

### Identification of highly variable proteins

Highly variable proteins^30^ were identified while considering variation between the cell lines by calculating a modified “quantile” standard deviation for each protein, ignoring the lowest and highest values for each protein. Then, the distribution of the modified standard deviation was modeled using a mixture of Gaussian distributions and we used an expectation-maximization method (EM) to estimate the different mixture components using the package mixtools, version 1.2.0. The EM process converged in a two distribution solution, which we assumed to represent the highly variable and the unmodulated proteins. Using this model, we estimated the number of highly variable proteins and selected a modified standard deviation threshold, which optimized the number of highly variable minus unmodulated proteins. As the EM process inevitably produces slightly different thresholds every time it is executed, we performed ten iterations and rounded the mean of the iterations to the lower 0.5 in order to have a reproducible solution.

### Clustering of proteomics data

We submitted three different datasets to the clustering procedure: the full panel of cell lines, B cells (BCP-ALL, B-ALL) only, and T cells (T-ALL) only. For each of these datasets, we identified the set of highly variable proteins as detailed above. We then marked samples that exhibited no Pearson correlation of 0.5 or greater to any other sample using all proteins with valid values only. Sample replicates were excluded. In order to identify the optimal number of clusters, we ran the consensus cluster algorithm using the R package ConsensusClusterPlus, version 1.52.0, applying the following parameters: pFeature =  0.8, pItem = 0.8, reps = 2000, clusterAlg = hc. We filtered the dataset for highly variable proteins and non-outlier samples and clustered with different distance measures (Pearson, Spearman) and linkages (average, ward.D2, complete). The number of clusters was determined by the elbow method and the delta area of the cumulative distribution function (Supplementary Fig. 3b). After having determined the number of clusters, we reestablished the original datasets with all proteins and the outlier samples and performed hierarchical clustering using 1 - Pearson correlation as distance measure and ward.D2 as a linkage method. Biological sample replicates were assigned to the cluster of their parents. Uncertainty of protein clusters were assessed by determining approximate bootstrap probabilities using pvclust R package (version 2.2-0).

### Consensus clustering of transcriptomics data

We applied the same method, followed for finding highly variable proteins, on RNA using log-transformed Transcripts Per Million (TPM) of only protein-coding RNAs. Highly variable protein-coding RNAs were utilized to find an optimal number of clusters using consensus clustering in a similar approach followed for protein data. RNA and protein clusters were compared using the Sankey plot implemented in the ggalluvial R package (version 0.12.3). Uncertainty of RNA clusters were assessed by determining approximate bootstrap probabilities using pvclust R package (version 2.2-0).

### Differential abundance analysis of proteomics data

Differential abundance of the proteomics data was performed using DEqMS, version 1.6.0^25^. When comparing samples within a specific ALL lineage (B, T), we only selected samples belonging to the same lineage. For each specific comparison, we first stratified the samples to be compared into two different groups. The remaining samples were passed in as a combined third group (“other”). Each of the three groups needed to have at least one valid quantification value for a respective protein. In parallel, for each protein, we calculated the sum of quantified PSMs per TMT set. Only sets contributing to the actual DEqMS analysis were taken into account. Then we computed the minimum PSM count across all of these sets while ignoring zero and NA values. If a protein had no quantified PSM, it was excluded from the analysis. This final PSM count per protein was then used to build the empirical Bayes statistics. The significance cut-off was set to an adjusted *P* value of 0.01 and fold changes to log2(1.5)-fold difference in abundance.

### Correlation analyses

Correlation between mRNA and protein levels for each gene was performed using overlapping genes (*n* = 8981) between the transcriptomic and proteomic data using 64 matched samples. Correlation between the mRNA and protein abundance values for each of these genes was determined using Spearman’s ρ correlation method. Correlation analysis between sDSS and protein abundance of all quantified proteins (*n* = 12,446) for the 43 cell lines with DSRT were also performed using Pearson Rank Correlation. We used the cor.test() function in R which uses t-distribution statistics to calculate the *P* value of the correlation.

### Gene set enrichment analysis

Gene set enrichment analysis (GSEA, https://www.gsea-msigdb.org/gsea/index.jsp) of gene lists from DEqMS, edgeR, and drug sensitivity correlation analysis were performed using the GSEA v4.1.0 software against priori-defined gene sets available at available from Molecular Signatures DataBase (MolSigDB). The priori-defined hallmark, Kyoto Encyclopedia of Genes and Genomes (KEGG), and Pathway Interaction Database gene sets were used. Weighted enrichment statistics was used for gene set sizes between 15 and 500, running with 1000 permutations.

### Complex regulation analyses

To investigate protein complex regulation, we used mRNA–protein correlations of all cell lines for CORUM complexes^32^ (Complete Dataset) as calculated above. We excluded proteins of the spliceosome, Nop56p-associated pre-rRNA complex, ribosome, and proteasome in order to avoid bias of large complexes. Subcellular localization (neighborhood level) were downloaded from ref. ^117^. For miRNA analysis, we downloaded miRNA–mRNA interaction data from^118^. The initial dataset (hsa_MTI.xlsx file) consists of 502,652 interactions that were filtered for “Functional MTI” support corresponding to 8157 unique interactions. These were further condensed to CORUM complex gene targets, allowing 3500 interactions between 572 miRNAs and 835 gene symbol-centric transcripts for downstream analysis. Degradation profiles were extracted from supplementary table S4 from^119^. Welch’s *t*-test and ANOVA were used for two- and multi-level comparisons, respectively, after converting correlations to *z*-scores. For the identification of significant mRNA-protein deviations, we devised a three-step approach:

1. RNA and protein data were preprocessed by averaging replicate cell lines and filtering to include genes with more than 1 TPM and proteins with non-NA values in at least half of the samples.
2. Stability scores for each gene symbol were derived as standardized residuals after regressing protein log2 ratios to RNA TPM values using locally estimated scatter plot smoothing (loess) function to account for lineage-specific transcript expression.
3. Significant stability was called for absolute standardized residuals greater than 3.

Significant cases were annotated for mutations (Cancer Dependency Map, DepMap, Broad (2021): DepMap 21Q3 Public. figshare. Dataset. 10.6084/m9.figshare.15160110.v2) and CORUM complex membership^32^ (Complete Dataset).

### UMAP of drug–protein correlations

Using the Pearson correlation coefficient results calculated as described above, a 2D matrix of values was assembled representing Pearson correlations to gene symbol-centric protein quantification values for each drug. Drugs were limited to only the screen results meeting a minimum sDSS threshold of 8 for at least one cell line (*n* = 336 drugs, *n* = 9786 proteins). A scaled, centered matrix was generated and used as input for a PCA using the Seurat package in R (https://cran.r-project.org/web/packages/Seurat/index.html). This PCA calculation was used to generate an elbow plot and a jack straw plot, also using functions in the Seurat package. Assessed using the elbow plot and jack straw plot to identify PCs with significant contribution to variance in the dataset, the number of principal components used as input for UMAP initiation was chosen to be 27. Using the uwot package (https://cran.r-project.org/web/packages/uwot/index.html), the Pearson correlation values were input for calculation of UMAP embedding for two dimensions, performed using the following key parameters: n_neighbors = 25, local_connectivity = 1, spread = 3.5, min_dist = 0.05, metric = “euclidean”, pca = 27, init = “normlaplacian”, nn_method = “annoy”. The 2D UMAP was added to the Seurat package as a dimensional reduction and plotted as a ggplot2 object (https://cran.r-project.org/web/packages/ggplot2/index.html) using the DimPlot function in Seurat.

### Differential correlation analysis

Differential correlation analysis^81^ was performed using the DGCA package (version 1.0.2), which was applied to Pearson correlations calculated between the sDSS for the drugs and proteins in BCP-ALL (B_Lineage), or in T-ALL (T_Lineage). The differential correlation design detected differentially correlated drug–protein pairs by lineage. No imputation was performed and NA and zero values were excluded. Permutation testing for *P* values was performed for *n* = 10 permutations. The classification was performed to characterize differential correlations by their directions and significance in each group, and all results regardless of classification were analyzed and ranked by *z*-score and significance.

### Statistical analysis of flow cytometry data

All quantification was performed using the BD FacsDiva software (BD Biosciences). For each experiment, mean DMSO-treated counts were obtained across technical replicates (per cell line, per day of the experiment), and this mean value was used to normalize counts to a ratio of viable treated cell count divided by viable mean DMSO cell count. Normalization operations, statistical tests, and data plotting were performed in R using the packages ggplot2 (https://cran.r-project.org/web/packages/ggplot2/index.html), and ggpubr (https://cran.r-project.org/web/packages/ggpubr/index.html). *P* values were obtained from an unpaired *t*-test between groups performed using the stat_compare_means function in ggpubr. Significance was validated in un-normalized raw counts data. Criteria for exclusion of acquired data was established prior to experimental data acquisition and analysis, and all analyzed wells were obtained from experiments where event counts over time remained constant throughout flow cytometry acquisition and where DMSO-treated controls from the same cell line culture and plate had more than 100 quantified viable cells per HTS collection, to ensure technical robustness of flow cytometry acquisition and cell line preparation.

### Statistics and reproducibility

No statistical method was used to predetermine sample sizes. No data were excluded from the analyses. The experiments were randomized based on the date of cell lines obtained and cultured as well as cytogenetic type and subtype. Investigator blinding to conditions and outcome assessment was not applicable. No data were excluded from analysis and all data met the quality control standards as described above. All of the statistical analyses were conducted using R (v.3.6.2 or higher), the drug sensitivity statistics of TMP269 which was conducted using Graphpad Prism 8, and the statistical analysis of the flow cytometry was conducted with Microsoft Excel T.TEST() function using a two-sided and homoscedastic calculation. Correlations and associated *P* values (Spearman and Pearson) were calculated with the R functions cor() or cor.test() which uses t-distribution statistics to calculate the *P* value of the correlation. Linear models built with the R function lm(). Two-sided, unpaired *t*-tests were performed using *t*.test() and two-sided Welch’s *t*-test using t.test() was performed for pairwise comparisons unless otherwise specified. For the multiple group comparisons analysis of variance (ANOVA) test was performed using the function anova(). Figure panels were created using base R graphics and ggplot2 (v.3.3.3) using ComplexHeatmap (v.2.2.0) R packages and Graphpad Prism^8^.

### Reporting summary

Further information on research design is available in the Nature Research Reporting Summary linked to this article.

## Supplementary information

Supplementary Information

Supplementary Data 1

Supplementary Data 2

Supplementary Data 3

Supplementary Data 4

Supplementary Data 5

Supplementary Data 6

Supplementary Data 7

Supplementary Data 8

Supplementary Data 9

Supplementary Data 10

Supplementary Data 11

Supplementary Data 12

Supplementary Data 13

Supplementary Data 14

Supplementary Data 15

Supplementary Data 16

Reporting Summary

## Source data

Source Data

## Data Availability

The mass spectrometry proteomics data have been deposited to the ProteomeXchange Consortium via the PRIDE partner repository with the dataset identifier PXD023662. Annotations of proteins were based on the Ensembl 99, GRCh38.p13 human genome assembly released 16th of January 2020. The raw RNA-seq data generated in this study have been deposited in NCBI’s Gene Expression Omnibus and are accessible through GEO Series accession number GSE168386. Publicly available transcriptomic data for a total of 417 leukemia samples including 18 cases with MEF2D-rearrangements from pediatric patients were obtained from The European Genome-phenome Archive (EGA) (Dataset ID: EGAD00001002704 and EGAD00001002692) after access was provided from St. Jude Children’s Research Hospital - Washington University Pediatric Cancer Genome Project data access committee (Contact email: PCGP_data_request@stjude.org). Raw viable cell count output from flow cytometry experiments are uploaded as.txt files in our GitHub repository: https://github.com/isabelle-leo/FORALL/tree/main/data/flow_cytometry. The raw data for EGAS00001001952 data are protected and are not available due to data privacy laws. All associated metadata for derived cell lines in the main dataset, including gender and age of patients, was assembled from publicly available or previously published sources. Source data are provided with this paper. Analyzed data can be browsed using our interactive shiny app: https://proteomics.se/forall. Source data are provided with this paper.

## Code availability

The code used to analyse the proteomics data and all code used to generate the figure panels is available on our GitHub repository: https://github.com/isabelle-leo/FORALL.
