# Supplementary material for: Integrative multi-omics and drug response profiling of childhood acute lymphoblastic leukemia cell lines
Source: Nat Commun. 2022 Mar 30;13:1691. doi: 10.1038/s41467-022-29224-5 (PMC8967900; doi:10.1038/s41467-022-29224-5)
Supplement: Supplementary file 1 — Supplementary Information [file 41467_2022_29224_MOESM1_ESM.pdf]

Supplementary figures 1-7 related to:

Integrative multi-omics and drug response profiling of childhood acute lymphoblastic leukemia cell lines.

Authors

Isabelle Rose Leo[1§], Luay Aswad[1§], Matthias Stahl[1§], Elena Kunold[1], Frederik Post[1,2], Tom Erkers[3], Nona Struyf[3], Georgios Mermelekas[1], Rubin Narayan Joshi[1], Eva Gracia-Villacampa[4], Päivi Östling[3], Olli P. Kallioniemi[3], Katja Pokrovskaja Tamm[5], Ioannis Siavelis[1], Janne Lehtiö[1], Mattias Vesterlund[1], Rozbeh Jafari[1*]

Affiliations

[1] Clinical Proteomics Mass Spectrometry, Department of Oncology-Pathology, Karolinska Institutet, Science for Life Laboratory, Tomtebodavägen 23A, 171 65 Solna, Sweden

[2] Institute of Plant Biology and Biotechnology, University of Muenster, Schlossplatz 7, 48149 Muenster, Germany

[3] Molecular Precision Medicine, Department of Oncology-Pathology, Karolinska Institutet, Science for Life Laboratory, Tomtebodavägen 23A, 171 65 Solna, Sweden

[4] Division of Gene Technology, School of Engineering Sciences in Chemistry, Biotechnology and Health, KTH, Science for Life Laboratory, Tomtebodavägen 23A, 171 65 Solna, Sweden.

[5] Department of Oncology-Pathology, Karolinska Institutet, J6:140 BioClinicum, Akademiska stråket 1, 171 64 Solna, Sweden

[§] These authors contributed equally.

[*] Corresponding author

Correspondence to: rozbeh.jafari@ki.se

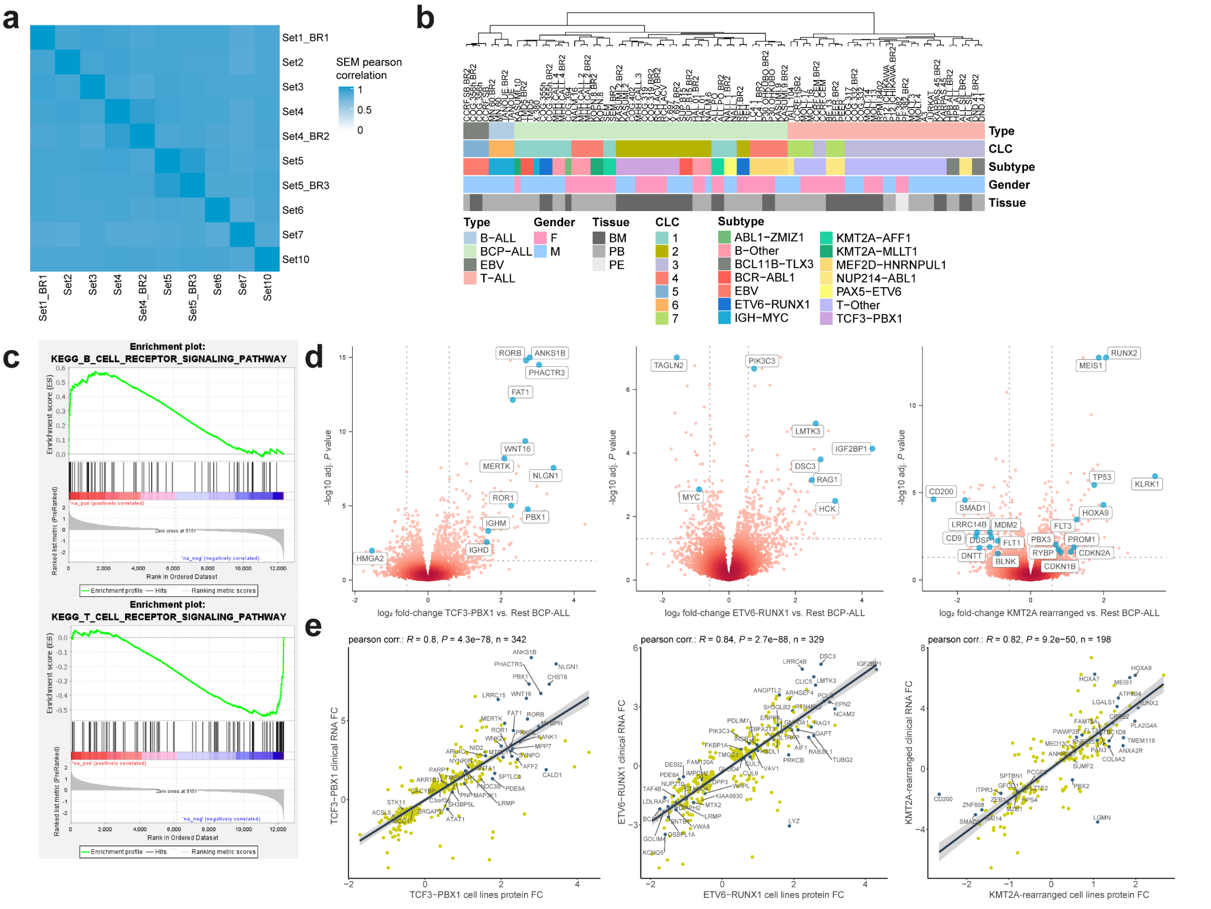

Supplementary Figure 1: Reproducibility of acquired data and agreement with clinical childhood ALL samples. (**a**) Pearson correlation heatmap of the biological replicates (n = 3) and technical replicates of SEM cell line biological replicate 1 (n=7) using the total overlapping proteins.BR; biological replicate. (**b**) Unsupervised Pearson Ward.D2 hierarchical clustering of the proteomics data and the biological replicates for selected cell lines (n = 32) that was generated approximately one year apart. BM bone marrow, PB peripheral blood, PE peripheral effusion. (**c**) GSEA plots of KEGG B-Cell (NES = 1.9, q-value = 0.0093) and KEGG T-Cell (NES = -1.9, q-value = 0.0093) using differentially abundant proteins between B-, and T lineage cell lines. The significance of NES was assessed by Kolmogorov-Smirnov statistics. (**d**) Volcano plots of differentially abundant proteins for the *TCF3-PBX1*, *ETV6-RUNX1*, *BCR-ABL1* and *KMT2A*-rearranged cell lines compared to the rest of the BCP-ALL cell lines using DEqMS with selected highlighted proteins in blue circles. The cut-off was set at q ≤ 0.01 and the fold changes (log2) was set to more than 0.58. Selected proteins are annotated as blue circles. (**e**) Fold changes of differentially expressed mRNA in clinical patient samples with *TCF3-PBX1, ETV6-RUNX1* and *KMT2A*-rearranged subtypes plotted against differentially abundant proteins in the *TCF3-PBX1, ETV6-RUNX1* and *KMT2A*-rearranged cell lines respectively. Only differentially expressed genes that passed a *P* value ≤ 0.01 are shown in the scatter plot with selected highlighted genes. N indicates the number of genes passing this criterion. *R* = Pearson correlation coefficient and *P* = t-distribution *P* value. The linear regression trendline (black) and its 95% confidence interval (shaded grey area) are shown in the graph.

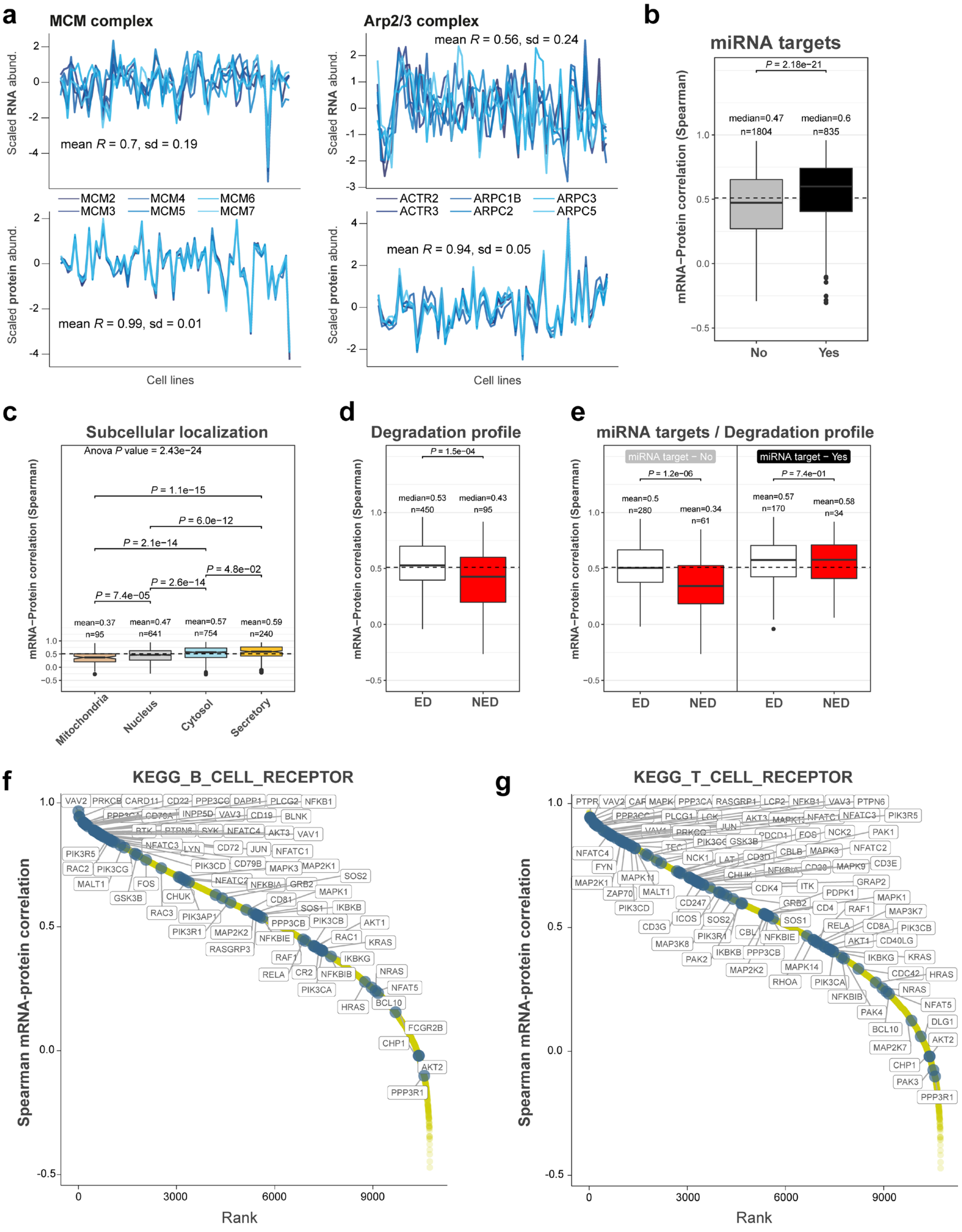

Supplementary Figure 2: Effect of protein complex formation, miRNA targeting, subcellular localization, protein degradation profile and cell lineage on ALL mRNA-protein correlations. (**a**) Line chart of protein and mRNA levels across the cell line samples for MCM and Arp2/3 protein complexes. The mean Spearman correlation coefficient (mean *R*) and standard deviation (sd) for the pairwise correlations of the complex members are shown

within each graph. (**b**) mRNA-protein Spearman correlation of miR- (n = 835) and non-miR-targeted (n = 1804) CORUM protein complex members. The top and bottom of the box reflect first and third quartiles with the median being represented by the band inside each box. The whiskers are 1.5 times the interquartile range and data beyond this range are plotted as individual points as outliers. Two-sided Welch's t-test was performed. (**c**) mRNA-protein Spearman correlation of CORUM protein complex members across subcellular locations ($n_{Mitochondria}$ = 95, $n_{Nucleous}$ = 641, $n_{Cytosol}$ = 754, $n_{Secretory}$ = 240, SubCellBarCode neighborhoods [1]). The top and bottom of the box reflect first and third quartiles with the median being represented by the band inside each box. The whiskers are 1.5 times the interquartile range and data beyond this range are plotted as individual points as outliers. One-way ANOVA for multi-group comparison was performed. Two-sided Welch's t-test for pairwise comparisons. (**d**) mRNA-protein Spearman correlation of CORUM protein complex members with exponential (ED, n = 450) and non-exponential (NED, n = 95) degradation. The top and bottom of the box reflect first and third quartiles with the median being represented by the band inside each box. The whiskers are 1.5 times the interquartile range and data beyond this range are plotted as individual points as outliers. Two-sided Welch's t-test with the *P* value (*p)*, indicated above the bracket. (**e**) mRNA-protein Spearman correlation of CORUM protein complex members with exponential (ED) and non-exponential (NED) degradation grouped by miR-target membership ($n_{miRNA\text{-}no/ED}$ = 280, $n_{miRNA\text{-}no/NED}$ = 61, $n_{miRNA\text{-}yes/ED}$ = 170, $n_{miRNA\text{-}yes/NED}$ = 34). The top and bottom of the box reflect first and third quartiles with the median being represented by the band inside each box. The whiskers are 1.5 times the interquartile range and data beyond this range are plotted as individual points as outliers. Two-sided Welch's t-test with the *P* value (*p*) indicated above the bracket. (**f**) Ranked mRNA-protein Spearman correlation of the genes in the KEGG_B_Cell_Receptor pathway highlighted in blue circles. (**g**) Ranked mRNA-protein Spearman correlation of the genes in the KEGG_T_Cell_Receptor pathway highlighted in blue circles. The y-axis in panel f and g shows the Spearman's rank correlation coefficient and the x-axis shows the Spearman correlation rank.

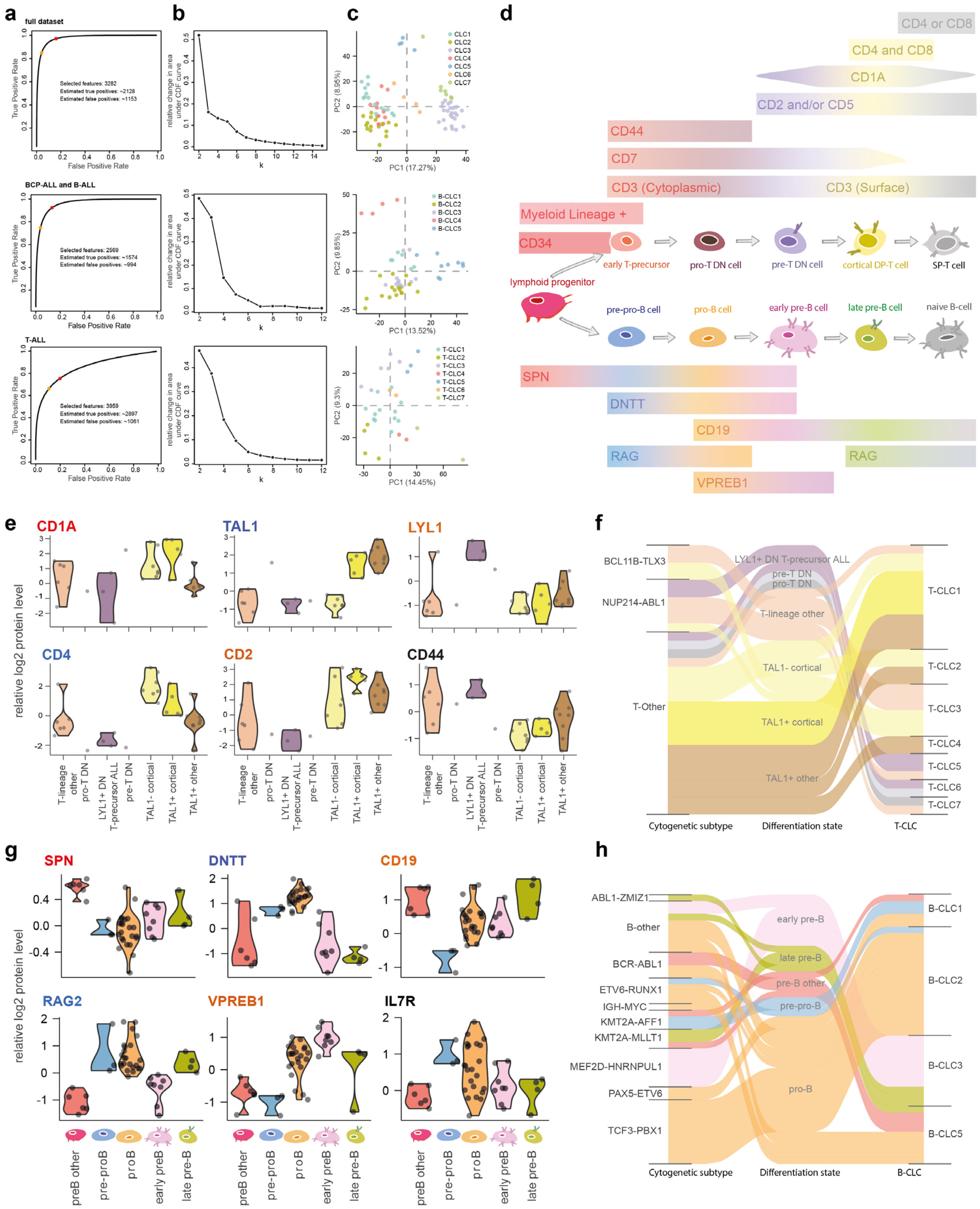

Supplementary Figure 3: Developmental staging of B- and T-lineages in childhood ALL cell lines. (**a**) Representative receiver operating characteristic (ROC) curves of the proteomics cluster analysis determining the highly variable proteins in all cell lines (top panel), B-lineage (middle panel) and T-lineage (bottom panel) cell lines separately (orange: best quantile for separation; red: rounded quantile used due to reproducibility). (**b**) Delta area curve of the consensus clustering analysis for all cell lines (top panel), B-lineage (middle panel) and T-lineage (bottom panel) cell lines separately. The y-axis shows the relative change in area under

the Cumulative Distribution Function (CDF) and the x-axis shows the number of clusters (*k*). (**c**) PCA plot of the first and second principal components of the determined CLC using highly variable proteins for all cell lines (top panel), B-lineage (middle panel) and T-lineage (bottom panel) cell lines separately. The colors represent the seven different CLC. (**d**) Schematic diagram of the T-ALL and BCP-ALL cell line differentiation stages, illustrating the markers chosen during the subgrouping assignment by cell state. Relative log2 protein levels of CD1A, TAL1, LYL1, CD4, CD2 and CD44 for T-ALL cell lines and DNTT, CD19, RAG1/RAG2, and VPREB1 for BCP-ALL cell lines by quantitative proteomics were assessed to immunotype cell lines. The T-ALL markers TAL1 and LYL1 were evaluated within DP or DN subtyped samples, respectively. TAL1+ cell lines which could not be identified by other cortical DP markers were classified as TAL1+ other. Markers indicated by triangular edges were excluded from the subtyping for these indicated stages. (**e**) Violin plots depicting the relative log2 protein levels of relevant T-cell markers across the T-ALL cell lines, grouped by assigned stage. (**f**) Sankey diagram illustrating the distribution and connections of T-ALL cell lines by T-CLC assignment (right), T-cell differentiation stage (center), and cytogenetic subtype (left). (**g**) Violin plots depicting the relative log2 protein levels of relevant BCP markers across the BCP-ALL cell lines, grouped by assigned stage. (**h**) Sankey diagram illustrating the distribution and connections of BCP-ALL cell lines by B-CLC assignment (right), B-cell precursor differentiation stage (center), and cytogenetic subtype (left).

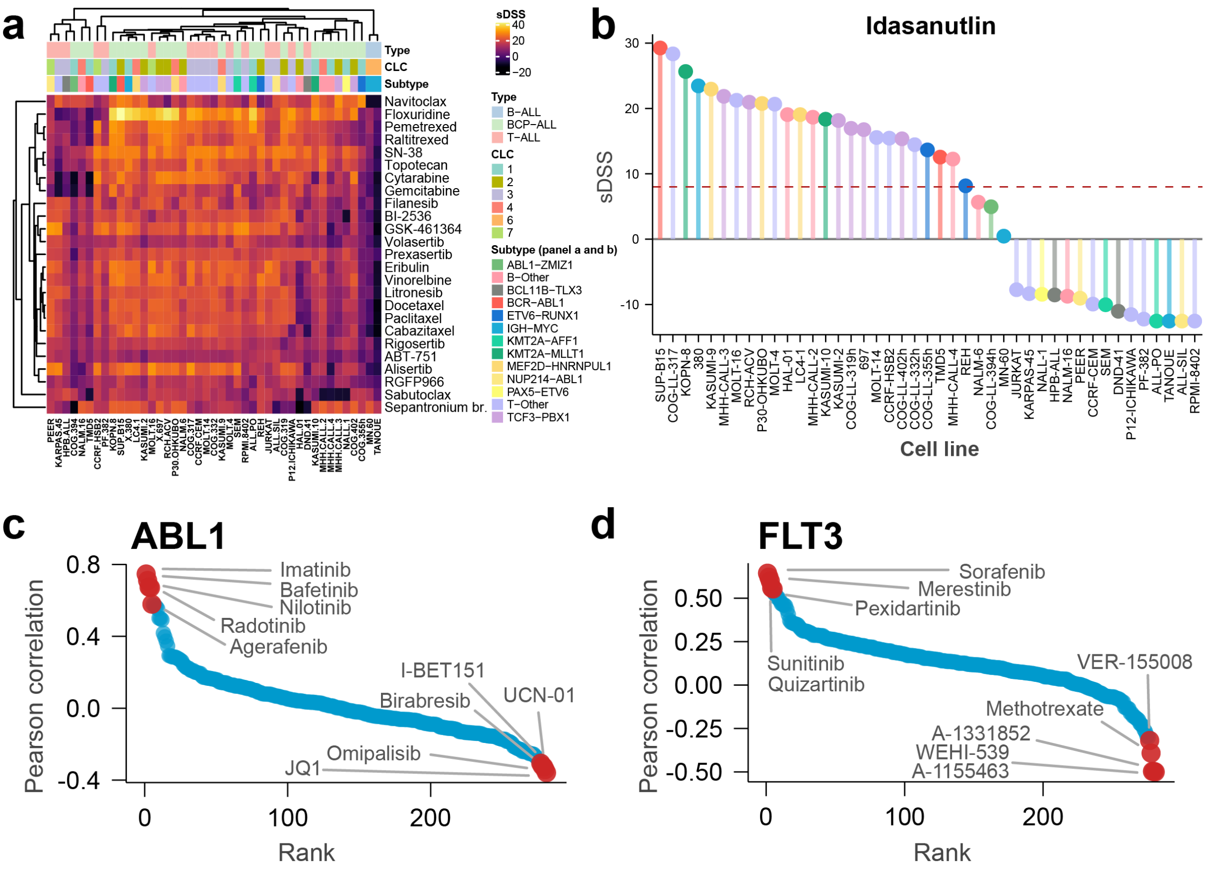

Supplementary Figure 4: Potency of highlighted drugs and relation to oncogenic drivers. (**a**) Heatmap showing the sDSS of selected potent drugs in the DSRT across the tested ALL cell lines. The x-axis is the cell lines ordered by the rank from hierarchical clustering (Pearson Ward.D2) and the y-axis is the individual sDSS for each drug. The legends indicate the CLC and cytogenetic subtype of the cell lines. (**b**) sDSS of the p53-MDM2 antagonist idasanutlin across the tested cell lines. The red dashed line indicates the selected threshold of sDSS = 8. The colors annotate the cytogenetic subtype of the cell lines illustrated in the panel a. (**c**) Pearson correlation of drug sensitivity and ABL1 protein levels. Top 5 positively and negatively correlating drugs are labeled in red. The x-axis shows the Pearson correlation coefficient and the y-axis shows the Pearson correlation rank. (**d**) Pearson correlation of drug sensitivity and FLT3 protein levels. Top 5 positively and negatively correlating drugs are labeled in red. The x-axis shows the Pearson correlation coefficient and the y-axis shows the Pearson correlation rank.

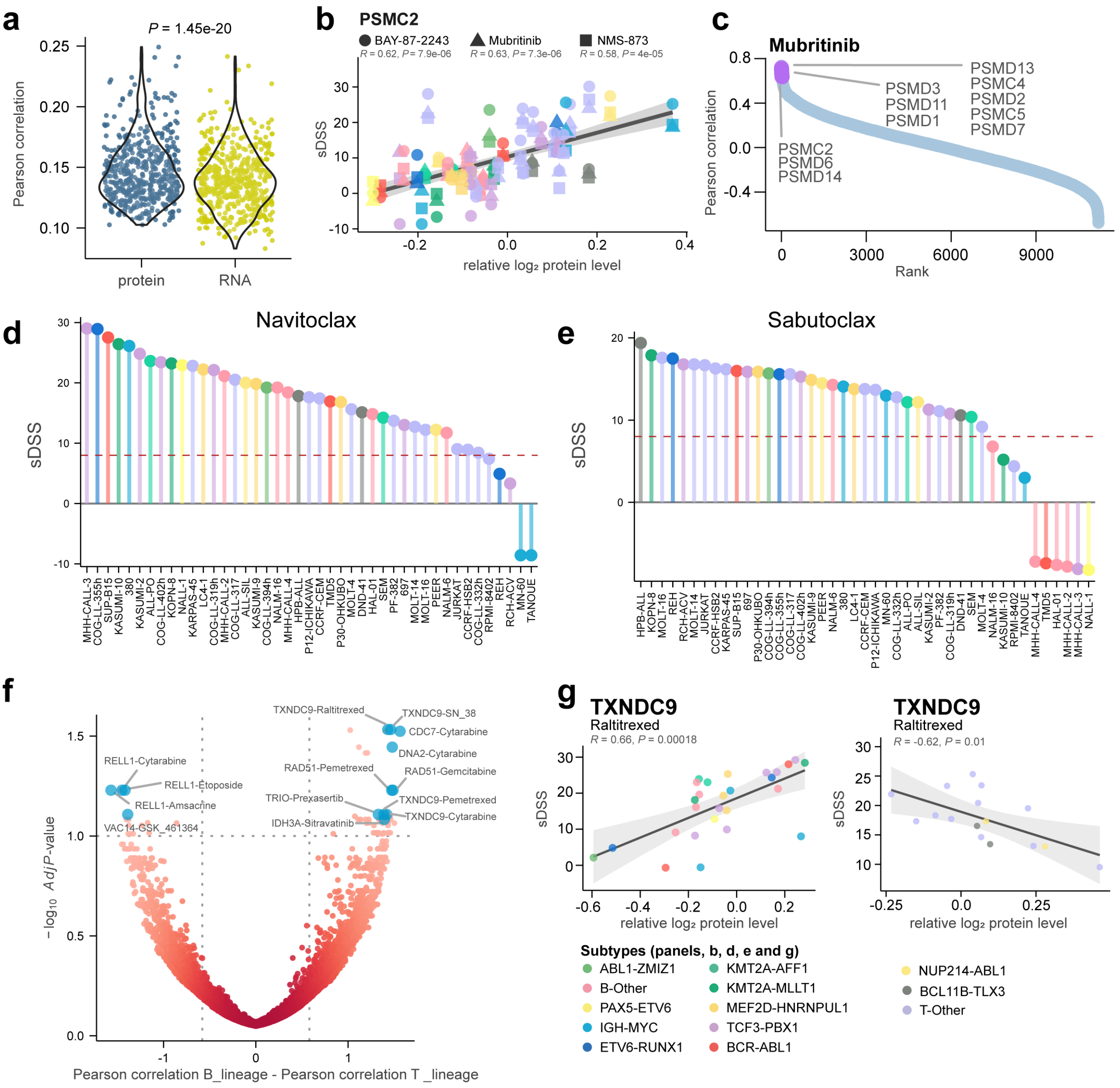

Supplementary Figure 5: Molecular profiling of lineage-specific drug mechanisms of action. (**a**) Comparison of Pearson average correlations of drug sensitivity per gene at the protein level (blue) or and the mRNA level (yellow) using a paired two-sided t-test. (**b**) Scatterplot of BAY-87-2243 (circle), Mubritinib (triangle), and NMS-873 (square) sDSS and PSMC2 protein levels. The x-axis shows the relative log2 protein level of PSMC2 and the y-axis shows the sDSS for each respective cell line (n = 43). *R* = Pearson correlation coefficient and two-sided *P* values of t-distribution (*p*), are shown within the concerned plot. The linear regression trendline (black) and its 95% confidence interval (shaded grey area) are shown in the graph. The colors indicate the cytogenetic subtype of the cell lines as in panel g. (**c**) Ranked Pearson correlations of sDSS and protein levels for Mubritinib with highlighted selected proteasomal subunits. (**d**) sDSS of the Navitoclax across the tested cell lines. The red dashed line indicates the selected threshold of sDSS = 8. (**e**) sDSS of the Sabutoclax across the tested cell lines. The red dashed line indicates the selected threshold of sDSS = 8. (**f**) Volcano plot for the DCA between the sDSS-protein correlation of B-lineage and T-lineage cell lines. X-

axis depicts the delta Pearson correlation coefficient between B-lineage and T-lineage. The cut-off was set at $q \leq 0.1$ and selected protein-drug pairs are annotated as blue circles. *P* values were derived from z-score distribution of differential correlation between the two analysis groups. (**g**) Scatterplot of raltitrexed sDSS and TXNDC9 protein levels in B-lineage (left panel) and T-lineage (right panel) cell lines. The x-axis shows the relative log2 protein level of TXNDC9 and the y-axis shows the sDSS for each respective cell line ($n = 43$). R = Pearson correlation coefficient. Two-sided t-distribution *P* values (*p*) are shown within the concerned plot. The linear regression trendline (black) and its 95% confidence interval (shaded grey area) are shown in the graph. The colors indicate the cytogenetic subtype of the cell lines.

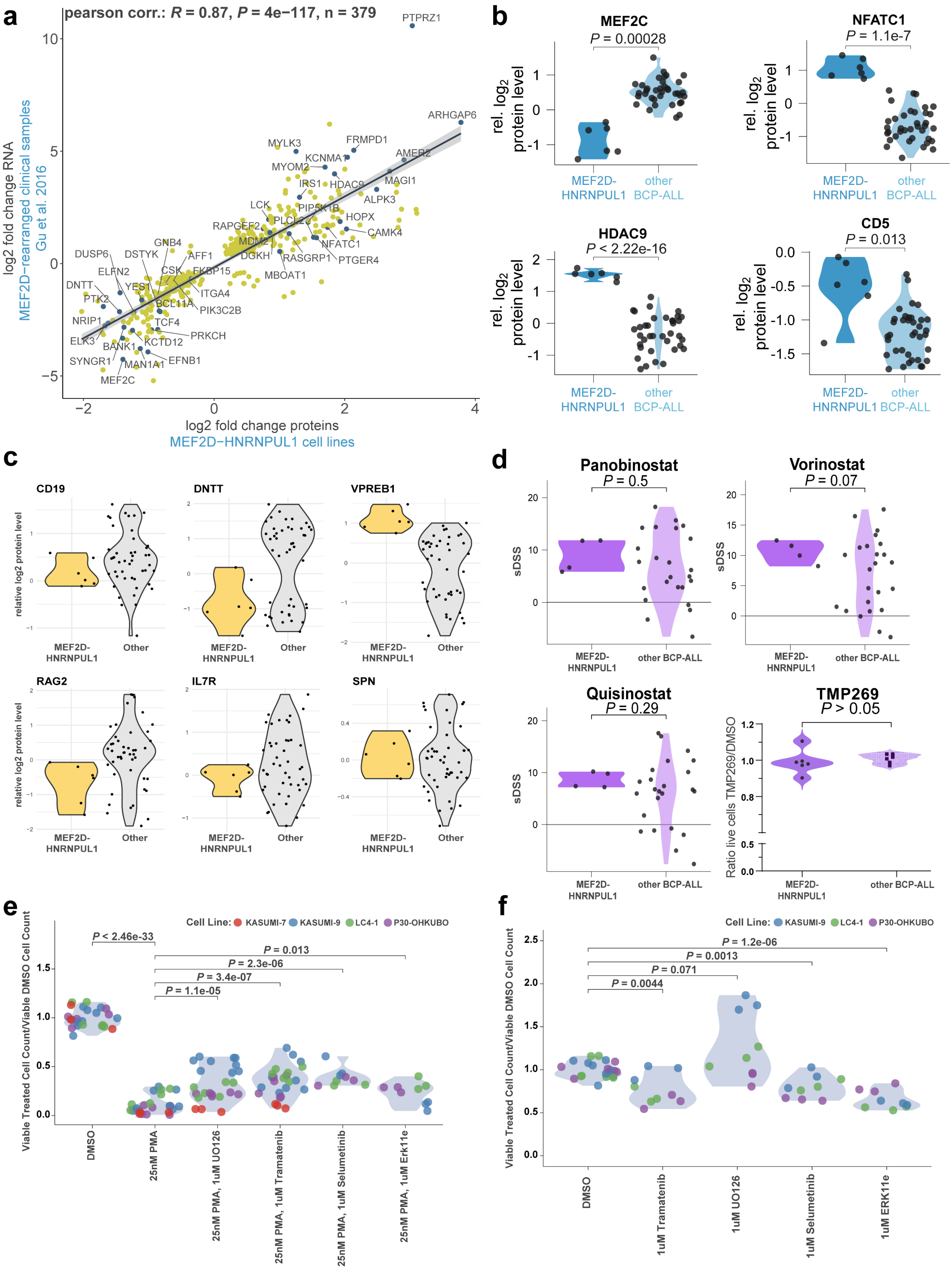

Supplementary Figure 6: Mechanistic profiling of drug sensitivity in MEF2D-HNRNPUL1 cell lines. (**a**) Fold changes of differentially expressed mRNA in *MEF2D*-rearranged samples from Gu et al. [2] study plotted against differentially abundant proteins in *MEF2D-HNRNPUL1* cell lines. Only differentially expressed genes that passed a *P* value ≤

0.01 are shown in the scatter plot with selected highlighted genes. N indicates the number of genes passing this criterion. *R* = Pearson correlation coefficient. *P* = two-sided t-distribution *P* value (**b**) Relative log2 protein levels of HDAC9, CD5, NFATC1 and MEF2C in *MEF2D-HNRNPUL1* cell lines compared to the remaining BCP-ALL cell lines. The *P* values were obtained from unpaired two-sided t-tests and indicated as *p*. (**c**) Immunophenotype of *MEF2D-HNRNPUL1* cell lines, showing relative protein levels for relevant markers, plotted alongside the relative protein levels for all other B-lineage ALL cell lines. (**d**) Comparison of drug sensitivity of selected HDAC inhibitors in *MEF2D-HNRNPUL1* cell lines vs other BCP-ALL cell lines. The x-axis shows sDSS. The *MEF2D-HNRNPUL1* cell lines were also not more sensitive to selective HDAC9 inhibitor (TMP269) at any of the six tested concentrations ranging from 0.0001 to 10µM in 10-fold dilution series, the x-axis for TMP269 plot shows the ratio of live cells between TMP269 and DMSO treated samples. The *P* values were obtained from unpaired two-sided t-tests and indicated as *p*. (**e**) Viable cell quantification normalized to corresponding mean DMSO viable cell count of *MEF2D-HNRNPUL1* fusion cell lines KASUMI-9, P30-OHKUBO, LC4-1, and KASUMI-7, treated with 25 nM of PMA alone or in combination with 1uM MEK inhibitors UO126, trametinib, or selumetinib. Alternatively, to block ERK directly, 1uM ERK inhibitor ERK 11e was used, and equal volume of DMSO was used as a control in triplicates. Viable cells were quantified by flow cytometry per 15uL HTS collection, excluding zombie aqua dyed non-viable cells. Results are merged from n=3 independent experiments. *P* values were obtained from unpaired t-tests. (**f**) Viable cell quantification normalized to corresponding mean DMSO viable cell count of *MEF2D-HNRNPUL1* fusion cell lines KASUMI-9, P30-OHKUBO, and LC4-1, treated with 1uM MEK inhibitors UO126, trametinib, or selumetinib, or 1uM ERK inhibitor ERK 11e. Equal volume of DMSO was used as a control in triplicates. Viable cells were quantified by flow cytometry per 15uL HTS collection, excluding zombie aqua dyed non-viable cells. Results are merged from three (n=3) independent experiments. *P* values were obtained from unpaired t-tests.

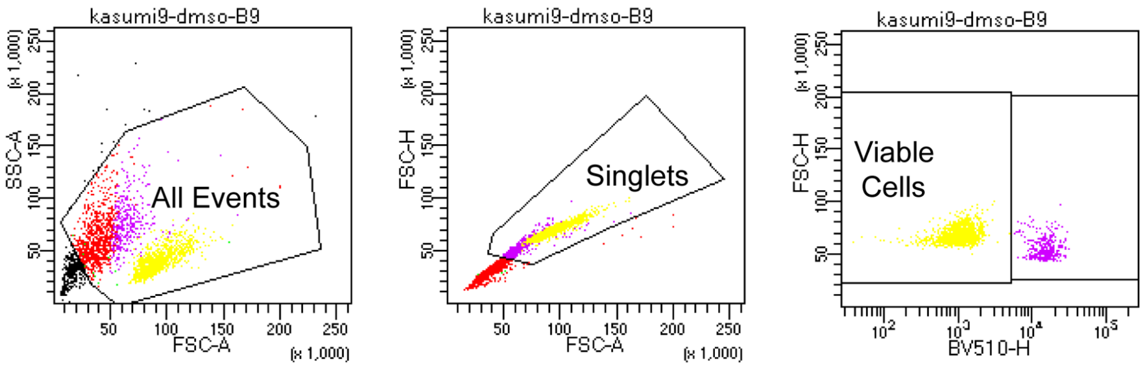

Supplementary Figure 7: Flow cytometry gating for all experiments was performed using the same gating scheme. All cells were stained using Zombie Aqua Live Dead stain (ThermoFisher), as previously described (Methods). Sequentially, events were first excluded outside forward scatter area/side scatter area (FSC-A/SSC-A) gate, next doublets were excluded outside the forward scatter area/forward scatter height (FSC-A/FSC-H) gate, and lastly events within the viable cells gate were exported for analysis, excluding dead cells positive in the BV510 channel. Viable cell counts were obtained using a BD Biosciences LSRFortessa flow cytometer, and cells were collected in equal volumes per well using the high throughput sampler (HTS) plate reader. Gating and quantification was performed using the BD FacsDiva software.

Supplementary references:

1. Orre LM*, et al.* SubCellBarCode: Proteome-wide Mapping of Protein Localization and Relocalization. *Mol Cell* **73**, 166-182 e167 (2019).

2. Gu Z*, et al.* Genomic analyses identify recurrent MEF2D fusions in acute lymphoblastic leukaemia. *Nat Commun* **7**, 13331 (2016).
