# Supplementary material for: Integrative multi-omics and drug response profiling of childhood acute lymphoblastic leukemia cell lines
Source: Nat Commun. 2022 Mar 30;13:1691. doi: 10.1038/s41467-022-29224-5 (PMC8967900; doi:10.1038/s41467-022-29224-5)
Supplement: Supplementary file 18 — Reporting Summary [file 41467_2022_29224_MOESM18_ESM.pdf]

nature research

Corresponding author(s): Rozbeh Jafari

Last updated by author(s): Jan 14, 2022

Reporting Summary

Nature Research wishes to improve the reproducibility of the work that we publish. This form provides structure for consistency and transparency in reporting. For further information on Nature Research policies, see our Editorial Policies and the Editorial Policy Checklist.

Statistics

For all statistical analyses, confirm that the following items are present in the figure legend, table legend, main text, or Methods section.

n/a	Confirmed	
☐	☒	The exact sample size (*n*) for each experimental group/condition, given as a discrete number and unit of measurement
☐	☒	A statement on whether measurements were taken from distinct samples or whether the same sample was measured repeatedly
☐	☒	The statistical test(s) used AND whether they are one- or two-sided<br>*Only common tests should be described solely by name; describe more complex techniques in the Methods section.*
☒	☐	A description of all covariates tested
☐	☒	A description of any assumptions or corrections, such as tests of normality and adjustment for multiple comparisons
☐	☒	A full description of the statistical parameters including central tendency (e.g. means) or other basic estimates (e.g. regression coefficient) AND variation (e.g. standard deviation) or associated estimates of uncertainty (e.g. confidence intervals)
☐	☒	For null hypothesis testing, the test statistic (e.g. *F*, *t*, *r*) with confidence intervals, effect sizes, degrees of freedom and *P* value noted<br>*Give P values as exact values whenever suitable.*
☒	☐	For Bayesian analysis, information on the choice of priors and Markov chain Monte Carlo settings
☒	☐	For hierarchical and complex designs, identification of the appropriate level for tests and full reporting of outcomes
☐	☒	Estimates of effect sizes (e.g. Cohen's *d*, Pearson's *r*), indicating how they were calculated

Our web collection on statistics for biologists contains articles on many of the points above.

Software and code

Policy information about availability of computer code

Data collection	Breeze version Master May 25th 2020 (https://github.com/potdarswapnil/Breeze); ImageJ software version 1.5Oi (https://imagej.nih.gov/ij/index.html); BD FacsDiva (https://www.bdbiosciences.com/en-us/instruments/research-instruments/research-software/flow-cytometry-acquisition/facsdiva-software); ProteoWizard tool suite (http://proteowizard.sourceforge.net/); MSGF+ v10072 (https://omics.pnl.gov/software/ms-gf); Percolator (v2.08); OpenMS project’s IsobaricAnalyzer (v2.0) (https://www.openms.de/openms220/); NGI pipeline (https://github.com/NationalGenomicsInfrastructure/ngi_pipeline);
Data analysis	cutadapt v 2.5 (https://cutadapt.readthedocs.io); STAR v 2.6.1b (doi: 10.1093/bioinformatics/bts635); featureCounts v 1.6.4 (http://subread.sourceforge.net/); ComBat-seq sva v 3.36.0 (DOI: 10.1093/nargab/lqaa078); edgeR v 3.32.1 (doi: 10.1093/bioinformatics/btp616); ConsensusClusterPlus v 1.50 (doi: 10.1093/bioinformatics/btq170); ggalluvial v 0.12.3 (https://cran.r-project.org/web/packages/ggalluvial/index.html); DEqMS v 1.6.0 (https://pubmed.ncbi.nlm.nih.gov/32205417/); GSEA v 4.1.0 (https://www.gsea-msigdb.org/gsea/msigdb/index.jsp); Seurat v 0.1.10 (https://cran.r-project.org/web/packages/uwot/index.html); uwot v 0.1.10 (https://cran.r-project.org/web/packages/uwot/index.html); ggplot2 v 3.3.3 (https://cran.r-project.org/web/packages/ggplot2/index.html); ggpubr v 0.4.0 (https://cran.r-project.org/web/packages/ggpubr/index.html), DGCA package (version 1.0.2) (https://cran.r-project.org/web/packages/DGCA/index.html), ComplexHeatmap (v.2.2.0) https://www.bioconductor.org/packages/release/bioc/html/ComplexHeatmap.html, The code used to analyse the proteomics data and all code used to generate the figure panels is available on our GitHub repository: https://github.com/isabelle-leo/FORALL.

For manuscripts utilizing custom algorithms or software that are central to the research but not yet described in published literature, software must be made available to editors and reviewers. We strongly encourage code deposition in a community repository (e.g. GitHub). See the Nature Research guidelines for submitting code & software for further information.

Data

Policy information about availability of data

All manuscripts must include a data availability statement. This statement should provide the following information, where applicable:

- Accession codes, unique identifiers, or web links for publicly available datasets
- A list of figures that have associated raw data
- A description of any restrictions on data availability

Data availability is stated in the paper in the “Data availability” section:
- The mass spectrometry proteomics data have been deposited to the ProteomeXchange Consortium via the PRIDE partner repository with the dataset identifier PXD023662
- The RNA-seq data discussed in this study have been deposited in NCBI's Gene Expression Omnibus and are accessible through GEO Series accession number GSE168386
- This study also makes use of data generated by the St. Jude Children’s Research Hospital – Washington University Pediatric Cancer Genome Project deposited at European Genome-phenome Archive (EGA) under the study accession code EGAS00001001952, https://ega-archive.org/studies/EGAS00001001952.
- Viable cell count data from flow cytometry experiments are hosted in our github repository: https://github.com/isabelle-leo/FORALL/tree/main/data/flow_cytometry
- Genesets used for GSEA has been obtained from: Molecular Signatures Database v7.5 https://www.gsea-msigdb.org/gsea/msigdb
- Analyzed data can be browsed using our interactive shiny app: http://proteomics.se/forall

Code availability is stated in the paper in the "Code availability" section:
- The code used to analyse the proteomics data is available and all code used to generate the figure panels is available on our github repository: https://github.com/isabelle-leo/FORALL

Field-specific reporting

Please select the one below that is the best fit for your research. If you are not sure, read the appropriate sections before making your selection.

☒ Life sciences ☐ Behavioural & social sciences ☐ Ecological, evolutionary & environmental sciences

For a reference copy of the document with all sections, see nature.com/documents/nr-reporting-summary-flat.pdf

Life sciences study design

All studies must disclose on these points even when the disclosure is negative.

Sample size	The number of cell lines included in the study (n=49) was chosen to include all accessible cell lines meeting the following criteria:<br>- Acute lymphoblastic leukaemia<br>- Childhood (age limit up to 20 years)<br>- B-ALL, BCP-ALL, or T-ALL lineage, derived from any tissue (bone marrow, peripheral blood) and not limited to any subtypes (genes fusions, mutations).<br>- Commercial availability or easily available upon request from repositories for better reproducibility.<br>- Commercial availability at the initiation of the project leading up to 49 childhood ALL cell lines.<br>- 49 cell lines are sufficient for statistical analyses.<br>Full information of the cell lines are available at supplementary table 1.
Data exclusions	Data exclusions were stated wherever needed in the text during various analyses to address certain questions applicable to specific subsets of the dataset, i.e. specific analyses by gene fusion phenotype or lineage. Non-protein coding transcripts were excluded from the analyses of RNAseq data. Quality control standards relating to viability of control samples were established prior to drug screening and flow cytometry analysis, as described in the methods section, and all presented experimental data adheres to these standards. No data were excluded from the analyses. No data was excluded from analysis and all data met the quality control standards as described above.
Replication	Our proteomics data is novel which limited our ability to validate some part of our results. We used public RNA-seq data from clinical samples to validate part of our results at transcriptomic and proteomics level, in the tested cases replication/reproducibility was successful. Replicate proteome profiles were obtained using DDA proteomics for n=32 cell lines, and using DIA proteomics for remaining cell lines, which all demonstrated robust reproducibility of proteome phenotypes in unbiased hierarchical clustering. Selected cell lines (n=16) were replicated in the transcriptomic dataset, where they all clustered together with their replicates. Flow cytometry expriments were replicated at minimum n=3 times, using separate cultures and drug treatments, as well as with a minimum of 3 biological replicate cell lines containing the same phenotype, and replications were successful. CETSA experiments were replicated twice where replication was successful.
Randomization	The cell line panel were chosen to represent as many possible known and rare subtypes of childhood ALL that could be obtained from a readily available source, selection of these cell lines was not altered based on additional randomization criteria. The experiments were randomized based on the date of cell lines obtained and cultured as well as cytogenetic type and subtype.
Blinding	The distinction of phenotypic and genetic fusion subtypes was confirmed in an unbiased way using unsupervised clustering. All conclusions were obtained by or supported by unbiased analyses of multi-omics data, which represented in-depth results obtained in a technically identical and unguided manner. Investigator blinding to conditions and outcome assessment was not applicable.

Reporting for specific materials, systems and methods

We require information from authors about some types of materials, experimental systems and methods used in many studies. Here, indicate whether each material, system or method listed is relevant to your study. If you are not sure if a list item applies to your research, read the appropriate section before selecting a response.

Materials & experimental systems

n/a	Involved in the study
☐	☒ Antibodies
☐	☒ Eukaryotic cell lines
☒	☐ Palaeontology and archaeology
☒	☐ Animals and other organisms
☒	☐ Human research participants
☒	☐ Clinical data
☒	☐ Dual use research of concern

Methods

n/a	Involved in the study
☒	☐ ChIP-seq
☐	☒ Flow cytometry
☒	☐ MRI-based neuroimaging

Antibodies

Antibodies used

HDAC1 (Thermo Fisher Scientific, cat. No PA1-860, RRID:AB_2118091), Phospho-ERK1/2 (Thermo Fisher Scientific, cat. No 14-9109-80, RRID:AB_2572925), ERK1/2 (Thermo Fisher Scientific, cat. No 13-6200, RRID:AB_2533024), b-actin (Santa Cruz Biotechnology Cat# sc-47778 HRP, RRID:AB_2714189). Primary Thermo Fisher Scientific antibodies were used at a dilution of 1:1000 and the Santa Cruz Biotechnology antibodies were used at a dilution of 1:500. (HRP)-conjugated secondary antibodies (lot 3208198, Abcam, cat no. AP127P for mouse anti-human ab and SCBT (sc-2005) for rabbit anti-human ab) used at a dilution of 1:5000.

Validation

All antibodies were tested by the manufacturer for relevant applications to ensure specific staining to the antigen without cross-reactivity. Advanced validation by provider sh/siRNA or knockout): HDAC1 (Thermo Fisher Scientific, cat. No PA1-860, RRID:AB_2118091), Phospho-ERK1/2 (Thermo Fisher Scientific, cat. No 14-9109-80, RRID:AB_2572925), ERK1/2 (Thermo Fisher Scientific, cat. No 13-6200, RRID:AB_2533024), b-actin (Santa Cruz Biotechnology Cat# sc-47778 HRP, RRID:AB_2714189).

Eukaryotic cell lines

Policy information about cell lines

Cell line source(s)

A3 ATCC; ALL-PO BBCF/iclc.it; ALL-SIL DSMZ; BE-13 DSMZ; CCRF-CEM DSMZ; CCRF-HSB2 DSMZ; CCRF-SB ATCC; COG-LL-317 COG; COG-LL-319h COG; COG-LL-332h COG; COG-LL-355h COG; COG-LL-356h COG; COG-LL-394h COG; COG-LL-402h COG; DND-41 DSMZ; HAL-01 DSMZ; HPB-ALL DSMZ; JURKAT DSMZ; KARPAS-45 Sigma; KASUMI-10 JCRB; KASUMI-2 DSMZ; KASUMI-9 JCRB; KOPN-8 DSMZ; LC4-1 JCRB; MHH-CALL-2 DSMZ; MHH-CALL-3 DSMZ; MHH-CALL-4 DSMZ; MN-60 DSMZ; MOLT-13 DSMZ; MOLT-14 DSMZ; MOLT-16 DSMZ; MOLT-17 DSMZ; MOLT-3 DSMZ; MOLT-4 DSMZ; NALL-1 JCRB; NALM-16 DSMZ; NALM-6 DSMZ; P12-ICHIKAWA DSMZ; P30-OHKUBO DSMZ; PEER DSMZ; PF-382 DSMZ; RCH-ACV DSMZ; REH ATCC; RPMI-8402 DSMZ; SEM DSMZ; SEM-BR-NOPS DSMZ; SUP-B15 ATCC; TALL-104 ATCC; TANOUE DSMZ; TMD5 JCRB; 380 DSMZ; 697 DSMZ.

Authentication

All cell lines were authenticated by STR profiling (Eurofins Genomics, Ebersberg, Germany). (Jan 2019).

Mycoplasma contamination

All cell lines were tested for Mycoplasma by MycoAlert Mycoplasma detection kit (Lonza). All cell lines used in this study tested negative for mycoplasma.

Commonly misidentified lines
(See ICLAC register)

The following cell lines from the ICLAC commonly misidentified lines register are included in the dataset: A3, BE-13. Their status as derivative cell lines is disclosed, and they have been characterized as derivative from cell lines which meet our cell line inclusion criteria. All metadata represents the identity of these cell lines based on their known derivative, and does not include misidentified characteristics.

Flow Cytometry

Plots

Confirm that:

☐ The axis labels state the marker and fluorochrome used (e.g. CD4-FITC).

☒ The axis scales are clearly visible. Include numbers along axes only for bottom left plot of group (a 'group' is an analysis of identical markers).

☐ All plots are contour plots with outliers or pseudocolor plots.

☒ A numerical value for number of cells or percentage (with statistics) is provided.

Methodology

Sample preparation	All cell lines were cultured assuspension cell lines and diluted to a plating density of 500k cells/mL. Cells were treated with soluble compounds at the stated concentrations for 72 hours in standard tissue culture incubation conditions (37C, 5% CO2) in a 96-well sterile tissue culture plate (Corning). All drug treatments and DMSO controls were brought to the same relative DMSO volume of 1:200. Following treatment, non-viable cells were stained using 1:500 Zombie Aqua Live Dead stain (ThermoFisher), diluted in PBS (Invitrogen) and added directly to the plated cells (1:2 volume). Cell staining was performed for 1.5 hours on ice, and during staining and all subsequent steps cells were protected from light using aluminum foil. No specific marker (e.g. CD4-FITC etc), dye were used to asses viability. Supplementary info 3 shows the Flow cytometry gating.
Instrument	BD Biosciences LSRFortessa flow cytometer with HTS plate reader
Software	BD FacsDiva
Cell population abundance	The relevant quantified cell populations were viable cells lacking Zombie Aqua Libe/Dead stain positivity, without assessment of surface markers or morphology.
Gating strategy	Gates were optimized to exclude noise by FSC-A/SSC-A, to exclude doublets by FSC-A/FSC-H, and to exclude dead cells positive in the BV510 channel.

☒ Tick this box to confirm that a figure exemplifying the gating strategy is provided in the Supplementary Information.
